# *Thymus serpyllum* Extract Improves Gastrointestinal Symptoms and Modulates Fecal Gut Microbiome Composition in a Multicenter, Randomized, Placebo-Controlled Clinical Trial

**DOI:** 10.3390/medsci14060611

**Published:** 2026-09-28

**Authors:** Raquel López-Zambrano, Jorge García-García, Laura López-Escánez, Teresa Vezza, Luckman Gbati, Rafael Palacios-López, Benita Martin-Castaño, Gonzalo Vargas-Castillo, Margarita Martínez-Zaldivar, Antonio M. Caballero-Mateos, Federico García, Eduardo Redondo-Cerezo, Raúl Fernández García, Emilio Fernández-Varón, Rocío Morón, Ivo Pischel, Cynthia G. Suarez, Alba Rodríguez-Nogales, María Elena Rodríguez-Cabezas, Julio Gálvez

**Affiliations:** 1 Centro de Salud “Salvador Caballero”, Distrito Granada-Metropolitano, 18012 Granada, Spain; raquel.lopez.zambrano.sspa@juntadeandalucia.es (R.L.-Z.); margarita.martinezzaldivar.sspa@juntadeandalucia.es (M.M.-Z.); 2 Instituto de Investigación Biosanitaria de Granada (ibs.GRANADA), 18012 Granada, Spain; jgarcia.51@ugr.es (J.G.-G.); e.lle197@go.ugr.es (L.L.-E.); gluckman@correo.ugr.es (L.G.); nomuvest@gmail.com (R.P.-L.); bemarcas@gmail.com (B.M.-C.); ogy1492@hotmail.com (A.M.C.-M.); fegarcia@ugr.es (F.G.); eredondoc@ugr.es (E.R.-C.); raulbalerma@gmail.com (R.F.G.); emiliofv@ugr.es (E.F.-V.); rmoronr@gmail.com (R.M.); merodri@ugr.es (M.E.R.-C.); jgalvez@ugr.es (J.G.); 3 Servicio Microbiología, Hospital Universitario Clínico San Cecilio, 18016 Granada, Spain; 4 Department of Pharmacology, Center for Biomedical Research (CIBM), University of Granada, 18071 Granada, Spain; 5 Servicio Digestivo, Hospital Universitario Virgen de las Nieves, 18014 Granada, Spain; 6 Centro de Salud Las Gabias, Distrito Granada-Metropolitano, 18012 Granada, Spain; vargocas27@gmail.com; 7 Department of Internal Medicine, Santa Ana Hospital, 18600 Motril, Spain; 8 CIBER de Enfermedades Infecciosas (CIBER-Infecc), Instituto de Salud Carlos III, 28029 Madrid, Spain; 9 Servicio Farmacia Hospitalaria, Hospital Universitario Clínico San Cecilio, 18016 Granada, Spain; 10 School of Pharmacy, Pharmacognosy and Phytotherapy, University College London (UCL), London WC1N 1AX, UK; info@drpischel.com; 11 Dr. Ivo Pischel Consulting, 53547 Rossbach, Germany; 12 Finzelberg GmbH & Co. KG, 56626 Andernach, Germany; cynthia.suarez-rizzo@finzelberg.com; 13 MartinBauer Nutraceuticals, 10040 Rivalta di Torino, Italy; 14 CIBER de Enfermedades Hepáticas y Digestivas (CIBER-EHD), Instituto de Salud Carlos III, 28029 Madrid, Spain

**Keywords:** fecal microbiome, gastrointestinal discomfort, gut–brain axis, gut microbiota, polyphenols, quality of life, *Thymus serpyllum* extract

## Abstract

Background/Objectives: Functional gastrointestinal discomfort is highly prevalent and affects up to 40% of the global population and remains challenging to manage due to its multifactorial nature. Usual interventions include dietary and lifestyle modifications, such as targeted phytochemical-based interventions. This multicenter, double-blind, randomized, placebo-controlled trial investigated the efficacy and exploratory microbiome-related effects of a standardized wild thyme extract (WThE) administered for 8 weeks on gastrointestinal symptoms, health-related quality of life, and fecal microbiome dynamics in adults without diagnosed organic gastrointestinal disease who reported gastrointestinal discomfort. Methods: A total of 178 adults without diagnosed organic gastrointestinal disease and with persistent gastrointestinal discomfort were randomized to WThE (*n* = 90) or placebo (*n* = 88); 131 participants had observed week-8 outcome data. Results: The analysis showed a greater reduction in overall gastrointestinal symptom burden with WThE than placebo, measured by the GSRS total score, with the largest exploratory domain-level differences observed in indigestion, constipation, and diarrhea-related symptoms. Improvements were also observed in health-related quality of life. At the microbiome level, no significant between-group differences were detected in α- or β-diversity; however, taxon-level analyses identified specific changes in fecal microbial composition, and predicted functional profiling suggested associated pathway-level differences. Moreover, the extract was well tolerated, with no product-related adverse events reported during the 8-week intervention. Conclusions: Overall, WThE reduced self-reported gastrointestinal symptom burden and improved selected quality-of-life outcomes in adults with persistent gastrointestinal discomfort, supporting *Thymus serpyllum* as a safe and well-tolerated botanical intervention linked to favorable modulation of the fecal microbiome and enhanced gastrointestinal function. ClinicalTrials.gov no: NCT06639126.

## 1. Introduction

Functional gastrointestinal discomforts are highly prevalent, affecting up to 40% of the global population and representing a substantial clinical and socioeconomic burden [1]. These disorders are characterized by recurrent sensations of abdominal discomfort, changes in motility including both constipation and diarrhea [2], as well as gut microbial dysbiosis [3]. Such imbalances can influence overall digestive comfort and well-being, often coexisting within the same individual and contributing to reduced quality of life and increased reliance on supportive care. Psychosocial factors, including stress and psychological health, are also recognized as key determinants in the development and persistence of these disorders [4]. Among these conditions, Irritable Bowel Syndrome (IBS) stands out as particularly relevant, due to its high prevalence and profound impact on daily functioning and healthcare costs. However, a significant proportion of individuals experiencing functional gastrointestinal discomfort do not meet the full diagnostic criteria for IBS according to Rome IV [1], yet they still suffer from symptoms that compromise their daily well-being. Therefore, evaluating gastrointestinal comfort through patient-reported outcomes in this non-clinical population has emerged as a scientifically valid and necessary approach to validate therapeutic strategies to manage these specific situations [5].

Despite advances in the understanding of its pathophysiology, therapeutic options remain limited and are often associated with variable efficacy and tolerability. This has led to increasing interest in adjunctive strategies targeting multiple pathophysiological mechanisms simultaneously, including modulation of gut microbiota composition and function. Thus, the systemic impact of these microbial communities is essential, as gut microbiota critically influence host digestion by metabolizing indigestible dietary components, synthesizing essential micronutrients, and modulating nutrient bioavailability through intricate biochemical pathways, thereby playing a pivotal role in maintaining intestinal homeostasis and preventing the onset and progression of functional gastrointestinal disorders [6,7,8].

In this context, dietary-derived bioactive compounds—particularly polyphenols—have emerged as potential modulators of host–microbiome interactions. Polyphenols can undergo extensive microbial biotransformation, generating metabolites with anti-inflammatory, antioxidant, and neuromodulatory properties, and may influence microbial community structure in a manner consistent with prebiotic-like effects, which promote digestive comfort, supporting gut motility, or help maintain gut barrier integrity [9]. Representative microbial metabolites arising from this biotransformation include phenolic acids (such as hydroxyphenylpropionic and hydroxyphenylacetic acids), phenyl-γ-valerolactones derived from flavan-3-ols, urolithins derived from ellagitannins, and equol derived from isoflavones. Several of these catabolites display better bioavailability and bioactivity than their parent compounds and have been linked to the prebiotic-like and anti-inflammatory effects attributed to dietary (poly)phenols [10,11].

Among plant-based strategies to support digestive comfort are whole foods such as kiwifruit, *Aloe vera*, Khorasan wheat (*Triticum turgidum* ssp. *turanicum*), and almonds, as well as plant-derived extracts from red pepper, *Mentha* spp., *Curcuma* spp., lemongrass, *Boswellia serrata*, ginger, and anise. These ingredients may promote digestive comfort and help regulate intestinal transit, demonstrating a favorable safety and tolerability profile [12].

Within this framework, the validation of plant-based supplements that are both safe and effective in supporting gastrointestinal wellness is an area of growing scientific interest. One such candidate is wild thyme (*Thymus serpyllum* L.), a perennial shrub native to Eurasia and North Africa, traditionally used as tea, spice, and in folk medicine practices, particularly to support gastrointestinal comfort [13], to relieve sensations of fullness and bloating, and to support normal digestion. The presence of non-volatile phenolic compounds, including flavonoids, polyphenolic acids, and triterpenes, may contribute to its spasmolytic effects on smooth muscle and its ability to support gastric secretion, thereby facilitating normal digestion.

In addition, recent research has revealed that polyphenols can also function as prebiotics, selectively promoting the growth of beneficial gut bacteria [14]. These compounds also exhibit antioxidant activity, positioning *T. serpyllum* extracts as potential natural sources of antioxidants for use in nutritional supplements or functional foods [15]. Among the beneficial taxa reported to be enriched by dietary polyphenols are *Bifidobacterium*, *Lactobacillus*, *Akkermansia muciniphila* and *Faecalibacterium prausnitzii*, together with polyphenol-converting genera such as *Gordonibacter* and *Eggerthella*; through this combined antimicrobial and prebiotic action, polyphenols can favour a more balanced microbial community [11,14].

Preclinical studies have demonstrated beneficial effects of a *T. serpyllum* extract in models of intestinal inflammation, IBS, and diet-induced metabolic syndrome in mice [13]. In all cases, the extract supported intestinal barrier integrity, helped modulate immune responses, and balanced gut dysbiosis. In these models, the extract reduced the colonic expression of pro-inflammatory mediators, including TNF-α, IL-1β and iNOS, helped preserve epithelial barrier markers (such as MUC-2, MUC-3, ZO-1 and occludin), attenuated oxidative stress, and partially restored microbial balance, increasing beneficial taxa such as *Lactobacillus* and *Bifidobacterium* [16,17]. These findings provide preliminary evidence of potential benefits and suggest possible mechanisms of action. More recently, a clinical trial in humans reported that the same *T. serpyllum* extract improved bowel regularity and beneficially modulated gut microbiota composition [18].

Taken together, these observations provide a rationale for evaluating *T. serpyllum* extract in adults with gastrointestinal discomfort, while the extent to which its clinical effects are mediated by the microbiome remains uncertain. This is particularly relevant given that gastrointestinal discomforts are strongly associated with imbalances in intestinal homeostasis, including impaired barrier function and microbial gut dysbiosis [3]. Consequently, botanical extracts with immunomodulatory and prebiotic properties may offer a promising approach to support gastrointestinal wellness, combining both efficacy and safety.

The present study was designed to evaluate the effects of a chemically characterized *T. serpyllum* extract on gastrointestinal symptom burden and health-related quality of life in individuals with functional gastrointestinal discomfort. In addition, we investigated its impact on gut microbiota composition and predicted metabolic function using shotgun metagenomic sequencing, with the aim of exploring potential mechanistic links between microbial modulation and clinical outcomes.

## 2. Materials and Methods

### 2.1. Test Compounds and Analytical Characterization

#### 2.1.1. Wild Thyme Extract and Placebo

The product investigated was a dry extract of Wild Thyme (*Thymus serpyllum* L.), 360GUT^®^, batch no.22000540. The extract is a brown-beige fine powder, readily soluble in water, and was stored at ambient temperature protected from moisture and light. It was prepared from the aerial parts of wild thyme obtained from Poland and manufactured by water extraction (DER 4–8:1) under Good Manufacturing Practice by Finzelberg GmbH & Co. KG, Andernach, Germany, as described on several own patents [19]. For the placebo, microcrystalline cellulose was used. Both capsules contained 600 mg of product and were similar in colour and appearance.

#### 2.1.2. Quantification of Rosmarinic Acid by High-Performance Liquid Chromatography with Ultraviolet Detection

The content of the principal phenolic marker, rosmarinic acid, was quantified by high-performance liquid chromatography (HPLC-UV) with detection at 320 nm. Chromatographic separation was performed on a reversed-phase C18 column (250 × 4.6 mm, 5 µm particle size) maintained at 50 °C. The mobile phase consisted of solvent A (water containing phosphoric acid, 999:1, *v*/*v*) and solvent B (acetonitrile containing phosphoric acid, 999:1, *v*/*v*), delivered at a flow rate of 1.0 mL/min. The gradient program was: 0 min, 95% A/5% B; 10 min, 60% A/40% B; 20 min, 45% A/55% B; 21 min, 10% A/90% B; 35 min, 10% A/90% B; and 36 min, 95% A/5% B. The injection volume was 10 µL and UV detection was performed at 320 nm.

A rosmarinic acid reference standard was dissolved in methanol to prepare a solution at 20 µg/mL. Approximately 80 mg of extract was accurately weighed and sonicated in 20 mL of water. Subsequently, 20 mL of methanol was added, and the solution was sonicated again. The sample was then diluted to 100 mL with methanol/water (50:50, *v*/*v*), centrifuged at 13,400 rpm for 3 min, and the supernatant was injected into the HPLC system. Rosmarinic acid was identified by comparison of its retention time with that of the authentic reference standard.

Quantification was performed by external standard calibration using peak-area measurements at 320 nm, and the rosmarinic acid content was expressed as percentage (*w*/*w*) of the dry extract. The analytical approach was based on previously reported HPLC methods for *T. serpyllum* and related Lamiaceae species, in which rosmarinic acid was quantified using reversed-phase C18 chromatography with UV detection at approximately 320 nm.

#### 2.1.3. Identification and Fingerprint of WThE Analysis by Thin-Layer Chromatography

The identity and chromatographic fingerprint of the WThE preparation were assessed by thin-layer chromatography (TLC) according to the European Pharmacopoeia monograph “Wild Thyme”. The extract sample was sonicated in methanol and the resulting solution was applied to silica gel F254 TLC plates. Chromatographic separation was performed using the conditions specified in the European Pharmacopoeia monograph. After development and air drying, the plates were derivatized as described in the monograph and examined under UV light at 365 nm.

Flavonoids and polyphenols were analysed using rosmarinic acid and rutoside as reference substances. The chromatographic profile was evaluated under UV light based on the characteristic zones specified in the monograph, including the green/blue fluorescent zone and yellow zone in the lower third, blue fluorescent zones in the middle third, and the blue rosmarinic acid zone in the upper third (Supplementary Figure S1).

To further confirm the identity of *T. serpyllum* and distinguish it from *T. vulgaris* and *T. zygis*, polar constituents were analysed by TLC using dichloroethane–glacial acetic acid (98%)–methanol–water (50:25:15:10) as the mobile phase (Supplementary Figures S2 and S3). Plates were derivatised with anisaldehyde reagent R and evaluated according to their characteristic chromatographic profiles.

The same chromatographic procedures were applied to different production batches to assess the consistency of the phytochemical fingerprint and, where applicable, to compare the test batch with a previously characterised production batch. The chromatographic profiles were evaluated qualitatively according to the presence, position and overall pattern of the characteristic zones.

#### 2.1.4. Analysis of the Volatile Fraction of WThE by Gas Chromatography

The volatile fraction of WThE was analyzed by gas chromatography (GC) according to the European Pharmacopoeia monograph “Thyme”. Essential oil obtained by hydrodistillation of Thymus samples was dried over anhydrous sodium sulfate, diluted in heptane, and analyzed on a fused-silica capillary column coated with a polyethylene glycol stationary phase. Separation was achieved using a temperature-programmed method, and analytes were identified by comparing their retention times with those of authentic thymol and carvacrol reference standards. Quantification was performed using the peak-area normalization method, and chromatographic system suitability was verified by adequate resolution between thymol and carvacrol peaks. Thymol and carvacrol were quantified to determine their relative abundance and ratio, which served as a chemotaxonomic criterion to distinguish *T. serpyllum* from *T. vulgaris/T. zygis* essential oils.

#### 2.1.5. Phytochemical Profiling of WThE by Liquid Chromatography–High-Resolution Mass Spectrometry

Additional phytochemical characterization was performed by liquid chromatography–high-resolution mass spectrometry (LC-HRMS) to support species differentiation and to confirm the presence of characteristic phenolic acids and flavonoids in WThE. Analyses were performed on an Acquity UPLC system (Waters) coupled to a hybrid LTQ Orbitrap Discovery mass spectrometer fitted with a heated electrospray ionisation (HESI) source operated in negative-ion mode. Separation used a C18 column with a mobile phase of water containing 0.1% formic acid (A) and acetonitrile (B) at a flow rate of 0.3 mL/min, applying the following gradient: 5% B (0–1 min), 5–100% B (1–15 min), 100% B (15–17 min), 100–5% B (17–17.5 min) and 5% B (17.5–20 min).

### 2.2. Study Design and Participants

#### 2.2.1. Study Design

The study was a multicenter, randomized, placebo-controlled, parallel-group, prospective nutritional intervention conducted in adults over 18 years of age. Participants were allocated in a 1:1 ratio to WThE or placebo and followed for 8 weeks. Between February 2024 and May 2025, 178 participants were randomized (90 to WThE and 88 to placebo). Participants, investigators, and outcome assessors were masked to treatment allocation, and the active and placebo capsules were identical in appearance and organoleptic characteristics. No interim efficacy analysis was performed; all analyses were conducted after database lock, with no alpha-spending or early-stopping rules.

#### 2.2.2. Sample-Size Calculation

Prior to study initiation, a priori sample size calculation was performed based on the primary outcome. A total of 127 participants were required to detect a clinically meaningful difference between groups on the GSRS total score, the primary outcome, assuming a two-sided significance level (α) of 0.05, 80% statistical power, and an anticipated attrition rate of 20% (EpiDat 4.2) [20]. The target difference was prespecified in the study protocol as the minimal difference considered clinically meaningful; in accordance with CONSORT 2025 reporting recommendations, the primary outcome, significance level, power and attrition assumption are reported here, and undocumented assumptions were not reconstructed from the observed treatment effect [21]. The final enrolled sample (*n* = 178) deliberately exceeded the calculated minimum (*n* = 127) in order to ensure a sufficient number of evaluable paired baseline and end-of-study fecal samples for the shotgun metagenomic substudy after quality control, and to provide a conservative margin against the high and variable attrition characteristic of functional gastrointestinal disorders. 

#### 2.2.3. Randomization and Blinding

The randomization sequence was generated and safeguarded by an independent investigator not involved in any other aspect of the trial. Random allocation was produced using a computer-generated schedule with permuted blocks of variable size to maintain group balance throughout recruitment. Given the multicenter design, randomization was stratified by study site. Allocation concealment was ensured through a centralized secure system accessible only after confirmation of participant eligibility. Study personnel at each center enrolled participants and obtained the corresponding assignment, while investigators, participants, and outcome assessors remained blinded to treatment allocation for the duration of the study. For blinding purposes, the placebo capsules were identical in appearance and organoleptic properties to those containing WThE but did not contain any active compounds.

#### 2.2.4. Inclusion and Exclusion Criteria

Eligible participants were male or female adults aged 18–75 years, with a body mass index (BMI) < 35 kg/m^2^ who reported persistent gastrointestinal discomfort but had no diagnosed organic gastrointestinal disease or IBS as defined by the Rome IV criteria; the presence of gastrointestinal discomfort was confirmed at screening as a condition for inclusion. Exclusion criteria were antibiotic use within the preceding three months; pregnancy or lactation; inflammatory bowel disease; celiac disease; hematological, autoimmune, or immunodeficiency disorders; abdominal surgery or radiotherapy affecting gastrointestinal function within the preceding six months; and current treatment with opioids, prokinetics, laxatives, antispasmodics, nonsteroidal anti-inflammatory drugs, or proton-pump inhibitors.

#### 2.2.5. Intervention

Participants were randomly allocated to receive either a supplement containing WThE (600 mg/day) or placebo (600 mg/day of microcrystalline cellulose), administered orally in capsule form once daily for eight weeks.

No specific dietary-control monitoring was implemented during the study period; participants were instructed to maintain their usual daily routines, and no intervention on habitual diet or physical activity was performed in order to minimize interference with their normal lifestyle.

#### 2.2.6. Study Assessments

The protocol encompassed an initial screening assessment, followed by an 8-week treatment period with two study visits scheduled at baseline and at the end of the intervention.

At screening, all participants underwent a medical examination that included clinical laboratory assessments (hematology and serum biochemistry, including glucose, lipid profile, and hepatic and renal parameters, determined by standard automated clinical-chemistry methods at the participating hospital laboratories), medical history, and measurement of vital signs (including blood pressure), to ensure that only individuals without clinically relevant abnormalities were enrolled.

At baseline and at week 8, participants completed two validated questionnaires: the Gastrointestinal Symptom Rating Scale (GSRS) and the Quality of Life Questionnaire for Functional Digestive Disorders (FDDQL).

The GSRS is a validated questionnaire that quantifies the intensity of digestive complaints experienced during the previous week [22]. It includes 15 questions grouped into 5 gastrointestinal symptom domains: abdominal pain (upper abdominal pain, hunger pains, and nausea), reflux (heartburn and acid reflux), indigestion (rumbling in the stomach, bloated, burping, and passing gas/flatus), constipation (constipation, hard stools, and a feeling of incomplete evacuation), and diarrhea (diarrhea, loose stools, and an urgent need to defecate). Each item is rated on a seven-point Likert scale, where 1 indicates “no discomfort at all” and 7 indicates “very severe discomfort”. The overall GSRS score therefore ranges from 15 (absence of symptoms) to 105 (maximal symptom severity).

The FDDQL is a 43-item questionnaire specifically designed to evaluate the impact of these conditions on eight domains related to various aspects of daily life and emotional well-being: daily activities, anxiety, diet, sleep, discomfort, coping with the disease, control of the disease, and the impact of stress [23]. Each item is rated on a five-point Likert scale. For every domain, as well as for the global score (calculated as the mean of all domains except the impact of stress), raw scores were transformed onto a 0–100 scale using the expression [(sum of item scores − number of items)/(4 × number of items)] × 100, where 4 corresponds to the maximum score attainable per item and the number of items is domain-specific (e.g., three items in the stress domain). On this scale, 0 denotes poor and 100 good quality of life, so that higher scores, and increases over time, reflect better health-related quality of life.

Safety was monitored throughout the study. Participants were asked to report any adverse event at each scheduled visit and at any time between visits, and all events were recorded in the case report form by trained study personnel, including onset, duration, severity, outcome, and suspected relationship to the investigational product.

### 2.3. Fecal Sample Collection and Microbiome Analysis

To assess changes in fecal microbiome composition, stool samples were collected from all participants at two time points: before the intervention (T0) and at the end of the study (8 weeks; T8).

Participants were instructed to collect the stool sample in the OMNIgene·Gut KIT (DNA Genotek, Ottawa, ON, Canada). The samples were collected by the researchers after a maximum of 48 h and stored at the study site at −70 °C until shipment to Novogene Europe (Novogene GmbH, Munich, Germany) for shallow shotgun metagenomic sequencing. For the metagenomic analyses, DNA was extracted from the fecal samples using the Qiagen AllPrep PowerFecal DNA/RNA Kit (Qiagen, Hilden, Germany). DNA concentration measurement, library preparation and shallow metagenomic sequencing library (SMS) were performed by Novogene Europe, generating approximately 2 Gbp per sample.

### 2.4. Statistical Analysis

Continuous variables were summarized as mean and standard deviation (SD), whereas categorical variables were expressed as counts and percentages. The primary confirmatory clinical efficacy outcome for the present statistical analysis was the change in gastrointestinal symptom burden, measured by the GSRS total score from baseline to week 8. Individual GSRS domain scores and health-related quality of life assessed using the FDDQL were considered supportive outcomes.

Secondary outcomes included GSRS domain scores and health-related quality of life assessed using the FDDQL domains. The primary analysis followed the intention-to-treat principle and included all randomized participants with an available baseline assessment.

Treatment effects were estimated using analysis of covariance (ANCOVA) models, with the post-treatment score at week 8 as the dependent variable, treatment group as the main independent variable, and the corresponding baseline score as a covariate. Adjusted mean differences between groups and their 95% confidence intervals (CIs) were derived from marginal model predictions.

Missing outcome data at week 8 were handled using multiple imputation by chained equations, generating 20 imputed datasets under a missing-at-random assumption. Imputation models included treatment groups and the corresponding baseline score as predictors, with predictive mean matching as the imputation method to preserve the observed range of the scales. ANCOVA models were fitted separately in each imputed dataset, and estimates were pooled across imputations using Rubin’s rules. Model assumptions were evaluated in each imputed dataset by visual inspection of residual plots and assessment of homogeneity of regression slopes. Intention-to-treat was pre-specified as the primary analysis, in accordance with the CONSORT recommendations, because it preserves the benefits of randomization and minimizes the attrition bias that may arise when the analysis is restricted to completers. It should be noted that multiply imputed values represent model-based approximations derived from the observed data under the missing-at-random assumption, rather than actual individual observations; accordingly, the imputed datasets are intended for the unbiased estimation of group-level treatment effects rather than for reconstructing individual participant trajectories.

The complete-case (per-protocol) analysis was therefore pre-specified as a supportive sensitivity analysis, and its results are reported alongside the intention-to-treat estimates for the primary outcome.

Pre-specified sensitivity analyses were conducted to evaluate the robustness of the results: (i) a per-protocol analysis restricted to participants who completed the 8-week follow-up with available outcome data, and (ii) a tipping-point analysis applied to the primary outcome to assess the robustness of its conclusions to plausible departures from the missing-at-random assumption. The consistency of treatment effects was explored across GSRS and FDDQL domains; these analyses were considered secondary and interpreted as supportive. All tests were two-sided, and a *p* value of less than 0.05 was considered to indicate statistical significance. Statistical analyses were performed using R software v4.4.2 (R Foundation for Statistical Computing, Vienna, Austria), with multiple imputation implemented using the *mice* package and marginal estimates obtained with the marginal effects package.

### 2.5. Bioinformatic Processing and Metagenomic Analysis

For DNA microbiota analysis, raw sequence quality was assessed using FastQC (v0.12.1) and summarized with MultiQC (v1.18). Low-quality reads and adapter sequences were removed using fastp (v0.24.1). Subsequently, host-derived reads were identified and removed using KneadData (v0.12.1) against the human reference genome hg38. Only high-quality non-host paired-end reads were retained for downstream taxonomic analyses.

Taxonomic profiling was performed using MetaPhlAn 4 (v4.0.6), which assigns reads to clade-specific marker genes for accurate microbial community composition analysis. Functional profiling was conducted using HUMAnN4, enabling the reconstruction and quantification of microbial metabolic pathways. Further statistical analysis of taxonomic profiles was conducted in R software (v4.4.2). For β-diversity comparisons (Bray–Curtis dissimilarity), permutational multivariate analysis of variance (PERMANOVA, Adonis function) was used to test for differences in community structure.

For comparisons of alpha diversity indices and microbial ratios between groups, a linear mixed-effects regression model adjusted for covariates was employed by using the R lme4 package (2.0-6). Differential abundance analyses of microbial taxa and functional pathways were performed using MaAsLin3 (1.2.0), adjusting for relevant covariates, and incorporating patient-level random effects.

Associations were considered statistically significant based on FDR-adjusted *q*-values < 0.05.

### 2.6. Ethics

The trial was approved by the Regional Ethics Committee (CEIM/CEI Provincial de Granada, Junta de Andalucía). Protocol version 1 was evaluated by the committee on 30 May 2023, and the favourable opinion was issued on 7 June 2023, before recruitment began on 1 February 2024.

The trial was subsequently registered at ClinicalTrials.gov (NCT06639126) and conducted in accordance with the CONSORT guidelines. All participants provided written informed consent before any screening procedure. The registered entry corresponds to this same prespecified protocol; any differences between the registry fields and the present report reflect the standardized format of the registry rather than changes to the study design, masking, sample size, or outcomes.

## 3. Results

### 3.1. Phytochemical Characterization of WThE

The phytochemical characterization of WThE was performed using complementary chromatographic techniques, including HPLC-UV, TLC, GC, and LC-HRMS. These analyses confirmed the identity and chemical consistency of WThE (batch 22000540).

Quantification by HPLC-UV showed a rosmarinic acid content of 2.65% (*w*/*w*) of the dry extract, corresponding to the principal phenolic marker of the preparation (Figure 1).

TLC profiling reproduced the characteristic zones described in the European Pharmacopoeia “Wild Thyme” monograph and allowed *T. serpyllum* to be distinguished from *T. vulgaris* and *T. zygis* (Supplementary Figures S1–S3). The TLC fingerprint of batch 22000540 was consistent with the reference profile and reproducible compared with the previous production batch 21011607. GC analysis of the volatile fraction yielded a thymol:carvacrol ratio of 1:1.2, clearly distinct from that observed in thyme essential oil (Supplementary Figure S4).

The corresponding thymol and carvacrol peak-area percentages were 6.1% and 7.4%, respectively, compared with 50.6% and 3.1% in Thyme essential oil, yielding ratios of 1:1.2 and 16:1, respectively.

LC-HRMS profiling further supported species differentiation and confirmed the presence of characteristic phenolic acids and flavonoids (Supplementary Figure S5). These findings further supported the chemical differentiation of Wild Thyme from conventional Thyme and provided complementary evidence for the chemical identity of the test preparation.

Together, the analytical results demonstrated that batch 22000540 exhibited the expected phytochemical characteristics of *T. serpyllum* and provided a chemically characterized test material for the clinical intervention.

### 3.2. Study Population and Baseline Characteristics

A total of 248 subjects were screened for eligibility, of whom 190 met the inclusion criteria and 178 were enrolled and randomly assigned to the placebo (*n* = 88) or WThE (*n* = 90).

After randomization, 9 participants did not receive the assigned intervention because they did not attend Visit 1 (WThE, *n* = 5; placebo, *n* = 4). A further 38 participants discontinued during follow-up (WThE, *n* = 20; placebo, *n* = 18); specific reasons for discontinuation were not documented in the supplied flow information. Thus, 47 randomized participants discontinued (placebo, *n* = 22 [25.0%]; WThE, *n* = 25 [27.8%]); reasons for discontinuation were not specified, but no discontinuation was reported as related to an adverse event or to the investigational product.

Consequently, 131 participants completed the 8-week study with available outcome data (placebo, *n* = 66 [75.0%]; WThE, *n* = 65 [72.2%]). However, all 178 randomized participants were included in the primary intention-to-treat analysis, with missing outcome data handled using multiple imputation. The CONSORT flow diagram is shown in Figure 2.

Baseline demographic, clinical, and biochemical characteristics were comparable between groups. The overall mean age was 43.1 years (SD, 14.4), and most participants were female (80.7%). Body mass index was within the normal range in both groups (overall mean, 24.0 kg/m^2^).

No clinically meaningful differences were observed between groups in baseline metabolic or biochemical parameters (Table 1).

### 3.3. WThE Administration Was Associated with Improvements in Gastrointestinal Discomfort in Adults with Persistent Gastrointestinal Discomfort

At week 8, participants receiving WThE showed a greater reduction in overall gastrointestinal symptom burden compared with placebo, as reflected by a larger decrease in the GSRS total score (estimated mean change from baseline, −16.46 [95% CI, −19.77 to −13.14] vs. −9.74 [−13.49 to −6.00]), corresponding to an adjusted between-group difference of −6.71 points (95% CI, −12.16 to −1.26; *p* = 0.017).

Domain-specific analyses consistently favored WThE. Statistically significant between-group differences were observed for acid reflux (difference, −0.38; 95% CI, −0.74 to −0.03; *p* = 0.034), nausea (−0.41; 95% CI, −0.82 to −0.00; *p* = 0.049), loose stools (−0.82; 95% CI, −1.48 to −0.16; *p* = 0.017), diarrhea (−0.55; 95% CI, −1.10 to −0.01; *p* = 0.045), rumbling in the stomach (−0.58; 95% CI, −1.13 to −0.03; *p* = 0.039), burping (−0.52; 95% CI, −1.00 to −0.04; *p* = 0.034), constipation (−0.89; 95% CI, −1.44 to −0.33; *p* = 0.002), hard stools (−0.79; 95% CI, −1.33 to −0.26; *p* = 0.004), and incomplete evacuation (−0.57; 95% CI, −1.13 to −0.01; *p* = 0.046) (Figure 3).

The remaining GSRS subscales did not show clear between-group differences and are detailed in Table 2.

Sensitivity analysis demonstrated that the per-protocol analysis, restricted to the 131 participants who completed the 8-week follow-up, yielded estimates consistent with the primary intention-to-treat analysis in both direction and magnitude across the primary and secondary outcomes (Supplementary Table S1). For the GSRS total score, the adjusted between-group difference was −6.75 points (95% CI, −11.34 to −2.15; *p* = 0.004). Furthermore, a tipping-point analysis examined the robustness of the primary outcome to departures from the missing-at-random assumption by applying a progressive penalty δ to imputed GSRS values in the WThE group (Supplementary Figure S6). The treatment effect remained statistically significant for penalties of up to 4.5 points (adjusted between-group difference, −5.20; 95% CI, −10.30 to −0.09; *p* = 0.046) and lost significance at δ = 5.0 (−5.06; 95% CI, −10.17 to 0.05; *p* = 0.052). Notably, the point estimate continued to favor WThE across the full range of δ values examined (0 to 10 points), with the adjusted between-group difference exceeding −3.67 points in the most conservative scenario.

### 3.4. WThE Administration Improves Quality of Life in Individuals with Persistent Functional Gastrointestinal Discomfort

Consistent with the observed symptomatic improvement, WThE produced a greater improvement in overall health-related quality of life compared with placebo at week 8 (estimated mean change from baseline, 14.4 [95% CI, 11.31 to 17.5] vs. 6.54 [3.47 to 9.6]), corresponding to a between-group difference of 7.87 points (95% CI, 3.56 to 12.17; *p* = 0.002). Participants receiving WThE also showed greater improvements in selected FDDQL domains with statistically significant between-group differences, particularly coping with the disease (mean difference in change from baseline, 19.60; 95% CI, 13.72 to 25.49; *p* < 0.001), control of the disease (20.21; 95% CI, 9.87 to 30.54; *p* = 0.001), and stress (28.27; 95% CI, 18.18 to 38.36; *p* < 0.001). No clear between-group differences were observed for other categories (Figure 4, Table 3).

### 3.5. WThE Supplementation Was Associated with Specific Changes in Fecal Microbiome Composition and Predicted Functional Pathways

Analysis of α- and β-diversity revealed no significant differences between the WThE and placebo groups at baseline or at the end of the intervention, indicating no detectable global difference in fecal microbial community diversity between treatment groups (Figure 5A,B).

Nevertheless, taxonomic analyses revealed differences in the relative abundance of specific bacterial taxa between treatment groups. Consistent with these findings, Venn diagram analysis identified a distinct taxonomic profile associated with WThE supplementation (Figure 5C), showing that the thyme group harbored 16.1% unique bacterial taxa. Notably, several of these taxa have been uniquely associated with beneficial roles in gut homeostasis, including members of the *Faecalibacterium* and *Blautia* genera and the *Lachnospiraceae* family (Supplementary Table S2 in Figshare) [24].

At the phylum level, *Bacillota* and *Bacteroidota* remained the predominant phyla in both groups throughout the intervention. Interestingly, WThE supplementation was associated with the maintenance of *Bacteroidota* whereas a decrease in this phylum was observed in the placebo group (Supplementary Figure S7). Notably, *Lentisphaerae* showed a significant reduction in the placebo group compared with WThE (*q* = 0.04; Figure 5D), indicating a treatment-dependent effect on its temporal dynamics. At the genus level, longitudinal modelling revealed treatment-dependent changes in relative abundance at the end of the intervention (Figure 6A). Specifically, WThE supplementation was associated with a significant increase in *Phocaeicola* (*q* < 0.01) and a reduction in *Anaerostipes* (*q* = 0.04) and *Anaerobutyricum* (*q* = 0.01), whereas the placebo showed the opposite pattern, including a decrease in *Roseburia* (*q* = 0.0047), *Phocaeicola* (*q* = 0.0047), *Bacteroides* (*q* = 0.02)*,* and *Alistipes* (*q* = 0.01), together with an increase in *Anaerobutyricum* (*q* = 0.02). In addition, prevalence-based analysis supported and refined these findings, indicating that WThE reduced the prevalence of *Anaerostipes* (*q* = 0.004), *Anaerobutyricum* (*q* = 0.01), and *Phocaeicola* (*q* < 0.01), while in the placebo group a decrease in *Alistipes* (*q* = 0.01) and *Anaerobutyricum* (*q* = 0.02) was accompanied by an increase in *Bacteroides* (*q* = 0.02) and *Phocaeicola* (*q* = 0.0047). Taken together, these results suggest that WThE not only modulates the relative abundance of key genera but may also contribute to stabilizing their distribution across samples, in contrast to the divergent temporal patterns observed under placebo. At the species level (Figure 6B), the analysis revealed a significant reduction in the relative abundance of *Anaerostipes hadrus* following WThE supplementation (*q* = 0.03). In contrast, the placebo group exhibited significant decreases in *Phocaeicola vulgatus* (*q* = 0.042), *Phocaeicola dorei* (*q* = 0.042), and *Bacteroides uniformis* (*q* = 0.04) after the intervention. Consequently, prevalence analysis further supported these patterns. *A. hadrus* was highly prevalent at baseline in the WThE group but showed a significant reduction in prevalence after treatment (*q* = 0.03). In contrast, placebo was also associated with a decrease in the prevalence of *P. vulgatus*, *P. dorei*, and *B. uniformis* (*q* = 0.04). The overall genus and species abundance profiles are provided in Supplementary Figures S8 and S9. Meanwhile, the full results of the exact statistical tests for phylum, genus, and species are also available in Figshare, Supplementary Tables S3, S4 and S5, respectively [24]. 

In addition, HUMAnN4 was used to infer microbial pathway potential from the metagenomic sequence data (Figure 6C). Functional pathway analysis identified differences in predicted microbial pathway abundance between groups. In the placebo group, at the end of the intervention, several metabolic pathways were significantly reduced, including folate transformation I (*q* = 0.02), L-histidine degradation III (*q* = 0.03) and I (*q* = 0.02), taxadiene biosynthesis (*q* < 0.01), L-arginine biosynthesis III (*q* = 0.041), and the superpathway of geranylgeranyl diphosphate biosynthesis II (*q* = 0.031), indicating a broad decline in key metabolic functions. In contrast, no significant reductions in these pathways were detected in the WThE group. L-citrulline metabolism decreased in the placebo group (*q* = 0.01) and increased in the WThE group (*q* = 0.037). These findings describe differences in predicted functional potential and do not establish changes in microbial metabolic output. The exact statistical test is available at Figshare, Supplementary Table S6 [24].

Finally, based on the species showing significant differential abundance at the end of the intervention in the WThE and placebo groups, Spearman correlation analyses were performed integrating microbial features with GSRS and QoL scores at the final time point (Figure 7).

For *P. vulgatus* and *P. dorei*, which were significantly reduced in the placebo group, distinct symptom-associated patterns were observed. *P. vulgatus* showed a significant negative correlation with constipation (*q* = 0.03), indicating that higher abundance was associated with lower symptom severity. In contrast, *P. dorei* was negatively correlated with acid reflux (*q* = 0.03), constipation (*q* = 0.04), diarrhea (*q* = 0.03), and loose stools (*q* = 0.02), suggesting that lower abundance was associated with worse gastrointestinal symptomatology. Notably, in the WThE group, where *P. vulgatus* abundance was maintained over time, higher levels of this species were positively associated with incomplete evacuation (*q* = 0.03) and loose stools (*q* = 0.03), indicating that its stability was still linked to inter-individual variation in symptom scores. Additionally, *A. hadrus*, which was significantly reduced following WThE supplementation, showed a negative correlation with acid reflux within the same group (*q* = 0.04), suggesting that lower abundance was associated with higher symptom severity for this specific outcome. Regarding quality of life, no overall differences were observed in correlation patterns across most taxa. However, *A. hadrus* showed a weak positive correlation with the total score in both the placebo (r = 0.18) and WThE (r = 0.19) groups, indicating that higher abundance was consistently associated with slightly worse quality-of-life outcomes, although effect sizes were modest. The exact statistical tests are available at Figshare, Supplementary Tables S7 and S8 [24].

Overall, these findings indicate that species-level changes induced by WThE and placebo were differentially associated with gastrointestinal symptom profiles, highlighting the complex relationship between microbial abundance dynamics and host symptomatology.

### 3.6. Adherence to the Intervention and Safety Assessment

Adherence to the intervention was monitored by capsule count at each study visit and reinforced through participant self-report. Participants were instructed to return all unused capsules, and compliance was calculated as the percentage of capsules consumed relative to those dispensed. Subjects were considered adherent when intake reached at least 80% of the assigned doses. WThE was well tolerated, and no product-related adverse events were spontaneously reported during the 8-week intervention period.

## 4. Discussion

This study represents the first 8-week, randomized, double-blind, placebo-controlled, multicenter, prospective nutritional intervention designed to evaluate the ability of a supplement containing WThE to support the normal function of the gastrointestinal tract and reduce the impact of gastrointestinal discomfort on quality of life. A dropout rate of 26% was observed (47 out of 178 participants), which is consistent with previous clinical trials in functional gastrointestinal disorders, where attrition rates typically range from 15% to over 40% due to the fluctuating nature of symptoms, high placebo responsiveness, and psychosocial burden associated with these conditions [25]. Importantly, discontinuation rates did not differ significantly between groups. While the tipping-point analysis supported the robustness of the primary finding under plausible small departures from the missing-at-random assumption, the effect became non-significant under more pessimistic scenarios. This reflects the inherent uncertainty introduced by the 26.4% loss to follow-up and should be considered when interpreting the magnitude of the treatment effect. A higher proportion of female participants were enrolled, which is consistent with epidemiological evidence indicating a greater prevalence of functional gastrointestinal disorders among women [1].

At study completion, participants receiving WThE exhibited greater reductions in gastrointestinal symptom burden, particularly in the domains of indigestion, constipation, and diarrhea, which were accompanied by a notably better perception of health status and a lower impact of gastrointestinal discomfort on daily routines, manifesting greater emotional resilience and self-confidence. As expected, the placebo group also showed some improvement after the intervention; however, this did not diminish the consistent benefits observed in the WThE group, supporting a treatment effect beyond the change observed in the placebo group. Placebo responses are well documented in gastrointestinal studies, with rates of up to 35–40% reported in inflammatory bowel disease and IBS [26], largely driven by patient perception and expectation.

The present findings are biologically consistent with the pharmacological profile previously described for *Thymus serpyllum*. Preclinical studies have reported anti-inflammatory and visceral analgesic effects of *T. serpyllum* preparations in experimental models relevant to IBS, while other studies have demonstrated antioxidant, antimicrobial, and dose-dependent spasmolytic activities in gastrointestinal models [13,27]. The consistency of clinical findings across several gastrointestinal domains also provides a plausible physiological context for the observed symptom profile. Relief of upper abdominal discomforts such as bloating, rumbling, and burping is compatible with improved gastric motility and gas handling, consistent with the traditional carminative and experimentally demonstrated spasmolytic properties of thyme polyphenols [27]. Likewise, the reduction in constipation severity and stool hardness suggests a potential regulatory effect on colonic transit and stool consistency, possibly involving modulation of smooth muscle tone or intestinal secretion, as reported for *Thymus* spp. preparations [28]. The concurrent improvement in diarrhea-related symptoms further supports the possibility of a homeostatic effect on bowel function, consistent with evidence that polyphenol-rich extracts can influence both hypo- and hypermotility through coordinated effects involving microbial metabolites, enteric neuromuscular signaling, and mucosal immune pathways [15]. Accordingly, existing preclinical evidence provides supportive context for these clinical findings rather than a definitive mechanistic explanation. 

WThE supplementation also improved health-related quality of life, particularly in domains related to stress, coping, and disease control. These findings are consistent with a possible contribution of gut–brain interactions, although they should not be interpreted as direct evidence of gut–brain axis modulation because neuroendocrine, inflammatory, and neural biomarkers were not measured. The gut–brain axis involves complex and bidirectional communication through neural, immune, endocrine, and microbial metabolic pathways, and its relevance to gastrointestinal symptoms and psychosocial outcomes has been increasingly recognized in IBS [29,30]. In this context, polyphenol-derived microbial metabolites have been proposed as potential mediators linking dietary polyphenols with host neurobiological processes, including inflammatory, oxidative, neurotransmitter-related, and neuroendocrine pathways [31]. Thus, the improvements in stress-, coping-, and disease-control-related FDDQL domains provide supportive clinical evidence of improved health-related well-being and a rationale for investigating gut–brain mechanisms in future studies, while the underlying molecular pathways remain to be established.

A key strength of the present study is the longitudinal fecal microbiome analysis, which identified specific taxonomic and predicted functional differences without evidence of broad ecological shifts. No significant differences were observed in α-diversity or β-diversity metrics between groups, indicating that overall fecal microbial richness, evenness, and community structure remained relatively stable. This finding is relevant in the context of gastrointestinal disorders, in which alterations in microbial composition have been reported, but findings regarding diversity and specific microbial signatures remain heterogeneous across studies and methodological approaches [32,33]. Intervention studies in IBS have likewise demonstrated changes in specific taxa despite limited or absent effects on global diversity, supporting the concept that clinically relevant microbial responses may occur at a more specific taxonomic or functional level rather than through wholesale restructuring of the microbial community [34]. Accordingly, the lower-level taxonomic and predicted pathway differences observed in the present study are best interpreted as specific associations rather than evidence of global microbiome remodeling.

An important interpretive consideration concerns the anatomical origin of the microbial signal. The microbiome analysis was based exclusively on fecal samples, which primarily reflect distal intestinal microbial communities and do not necessarily capture microbial or physiological events occurring in the upper gastrointestinal tract or small intestine. Indeed, it has been argued that the healthy small intestine is largely a low-biomass environment without a stable resident microbiota, and that colonic or fecal communities should not be used as direct proxies for the small-intestinal environment [35,36]. Because the extract is ingested, its constituents and metabolites encounter the stomach and small intestine before reaching the colon; consequently, part of the observed symptomatic benefit could arise from direct, pre-colonic actions of thyme polyphenols, including spasmolytic effects on smooth muscle and effects on epithelial, secretory, sensory, neuroendocrine, or mucosal-immune signaling, rather than exclusively from changes in the fecal microbiota. Within this framework, the fecal-microbiome changes reported here are best interpreted as one downstream compartment that may represent a consequence, a correlate, or a mediator of the clinical response, and the terms “gut microbiota” and “microbiome remodeling” should accordingly be understood as referring specifically to the fecal (predominantly colonic) compartment.

From a taxonomic perspective, and consistent with the absence of changes in α- and β-diversity, WThE effects were restricted to specific taxonomic levels identified through longitudinal modelling. At the phylum level, WThE appeared to prevent the temporal decline observed in the placebo group for *Bacteroidota* while significantly modulating *Lentisphaerae*, suggesting an effect on compositional stability rather than expansion or depletion of major community components. The modulation of *Lentisphaerae* may be of interest in the context of gut physiology because members of this phylum have been associated with microbial pathways related to short-chain fatty acid metabolism, including acetate and formate production [37]. However, taxonomic abundance does not demonstrate metabolite production, and short fatty acids (SCFAs) were not measured in the present study. Therefore, the observed Lentisphaerae change should be interpreted as an alteration in microbial composition with potentially relevant functional capacity, rather than as evidence of increased SCFA concentrations. SCFAs are broadly implicated in mucosal immune regulation and intestinal homeostasis [38], but whether changes in Lentisphaerae in this trial translated into altered SCFA exposure remains unknown.

When evaluating differences at the genus level, the placebo group presented a reduction of several commensal genera, including *Roseburia* and *Alistipes*, alongside an increase in *Anaerobutyricum*. In particular, *Alistipes* has been consistently associated with protection against intestinal inflammation and maintenance of gut homeostasis [39]. Similarly, *Roseburia* contributes substantially to butyrate production, a metabolite essential for epithelial integrity and anti-inflammatory signaling in the colon [40]. In contrast, WThE supplementation was associated with the preservation of key commensals such as *Phocaeicola* and a reduction in *Anaerobutyricum*. The interpretation of changes affecting *Anaerobutyricum*, however, is not straightforward. Although reduced abundance of this genus has been reported in inflammatory bowel disease, increased levels have been described in other inflammatory conditions, including rheumatoid arthritis, suggesting that its biological significance is highly context dependent [41]. Considering that all participants in the present study presented baseline digestive symptoms, the reduction observed following WThE supplementation may reflect a selective ecological remodeling rather than a universally beneficial or detrimental effect. Focusing on *Phocaeicola*, the placebo group showed a decline in species such as *P. vulgatus*, *P. dorei* and *Bacteroides uniformis* taxa that play important roles in polysaccharide fermentation, SCFA production, and maintenance of gut homeostasis. Their depletion has been associated with impaired barrier function and inflammatory conditions, and in the present study this reduction was accompanied by a less favorable gastrointestinal symptom profile [42,43]. Conversely, the preservation of *P. vulgatus* following WThE supplementation may indicate a protective effect on microbial functions relevant to intestinal health. Additionally, a similar context-dependent interpretation may apply to *Anaerostipes hadrus*. Although this butyrate-producing species is generally considered a marker of a healthy microbiota [44], its expansion has also been linked to aggravated inflammation under dysbiotic conditions [45]. Therefore, the reduction observed after WThE supplementation may not necessarily represent a loss of beneficial bacteria, but rather a selective restructuring of the microbial community.

Beyond taxonomic changes, functional pathway analysis provided further insight into the biological relevance of the microbial shifts observed during the intervention. The placebo group exhibited a reduction in several metabolic pathways, particularly those involved in folate metabolism and amino acid processing. These pathways play important roles in epithelial renewal, immune regulation, and overall metabolic homeostasis, suggesting a possible reduction in functional potential over time [46]. In contrast, WThE supplementation was associated with preservation of microbial functions linked to intestinal homeostasis, most notably L-citrulline metabolism. Citrulline is closely related to intestinal epithelial function and arginine biosynthesis and has been implicated in the maintenance of barrier integrity and modulation of immune responses under conditions of physiological stress [47]. Collectively, these taxonomic and functional findings indicate treatment-associated differences in selected fecal microbial taxa and predicted pathways, without evidence of a global change in α- or β-diversity. Importantly, these pathway differences represent predicted functional potential and should not be interpreted as demonstrated preservation of metabolic activity, metabolic flux, or metabolite production.

The relationship between dietary polyphenols and the fecal microbiome provides an additional framework for interpreting these findings. Polyphenols can undergo extensive microbial biotransformation into lower-molecular-weight phenolic metabolites, while changes in microbial ecology may also influence microbial metabolites such as SCFAs. These metabolites and microbial products can influence intestinal barrier integrity, immune responses, metabolic regulation, and gut–brain signaling [48]. In this context, the specific taxonomic changes and predicted pathways observed following WThE supplementation are compatible with selective modulation of microbial functions rather than broad ecological restructuring. This interpretation is consistent with previous reports showing that the same WThE and its polyphenols promote the expansion of SCFA-producing bacteria and favorable microbial configurations [18,49].

The phytochemical composition of WThE provides a further mechanistic layer linking the extract to these microbiota-related observations. Rosmarinic acid was the principal quantified phenolic marker of the extract (2.65% of the dry extract), and this compound can undergo extensive microbial biotransformation in the human gut into lower-molecular-weight phenolic metabolites with potential physiological relevance. In work specifically addressing thyme phenolics, human faecal microbiota extensively catabolized rosmarinic acid and eriodictyol, with 3-(4-hydroxyphenyl)propionic acid identified as a major metabolite. Moreover, intake of thyme phenol-enriched olive oil increased faecal hydroxyphenylpropionic and phenylpropionic acids, providing direct human evidence that thyme-derived phenolics undergo substantial microbial transformation [50]. These findings are especially relevant because the study intervention utilized a chemically characterized WThE, with rosmarinic acid identified as its primary quantified phenolic marker.

Evidence from other Lamiaceae preparations further supports the plausibility of this metabolic route. Human data obtained with rosemary tea, another rosmarinic-acid-rich Lamiaceae preparation, demonstrated extensive microbial metabolism of ingested phenolics, with dihydrocaffeic acid and its phase-II derivatives among the main products identified [51]. Likewise, human fecal fermentation of flavonoid derivatives generated 3-(4-hydroxyphenyl)propionic acid, 3-phenylpropionic acid, and phenylacetic acid [52]. Collectively, these findings support the concept that the biological effects of polyphenol-rich botanical preparations may depend not only on their parent compounds but also on their transformation by the intestinal microbiota into smaller, potentially bioactive metabolites. Thus, the rosmarinic acid content identified in WThE provides an additional plausible link between the phytochemical composition of the extract, its interaction with the fecal microbiome, and the observed clinical effects. These compounds are literature-supported candidates rather than confirmed metabolites in the present study; consequently, targeted or untargeted metabolomic profiling is required to verify their formation and establish any relationship with the clinical response.

The microbiota-related interpretation is further supported by the direct biological activities previously attributed to the same WThE. Preclinical studies have shown that WThE exerts anti-inflammatory and antioxidant effects by downregulating key pro-inflammatory mediators such as TNF-α, IL-1β, and IL-6, while enhancing expression of the anti-inflammatory cytokine IL-10 [16,17]. Furthermore, the reduction in abdominal discomfort reported by participants is consistent with the visceral analgesic effects of WThE previously characterized in experimental models of IBS, where the extract significantly reduced visceral hypersensitivity. This effect has been linked to preservation of intestinal barrier integrity, as WThE prevents degradation of tight-junction proteins and reduces colonic oxidative stress [17]. These experimental findings provide biological plausibility for the clinical effects observed in the present trial and suggest that microbial modulation may act alongside direct effects of the extract on intestinal homeostasis. Overall, the clinical, taxonomic, functional, and phytochemical findings support a multifaceted model in which WThE may influence gastrointestinal symptoms through complementary microbial and host-mediated processes. Selective preservation of commensal taxa and predicted microbial pathways, together with the established capacity of the gut microbiota to transform rosmarinic acid and related thyme phenolics, may contribute to an intestinal environment associated with epithelial integrity, immune balance, and metabolic homeostasis. These microbiome-related processes may act in concert with the known spasmolytic, carminative, anti-inflammatory, antioxidant, barrier-protective, and visceral analgesic properties of thyme-derived compounds. Such a multifaceted mode of action is particularly relevant in functional gastrointestinal disorders, where visceral sensitivity, epithelial integrity, low-grade inflammation, microbiota–host communication, and gut–brain signaling are closely interconnected and may converge to influence symptom generation and severity.

The microbiome findings should nevertheless be interpreted at the appropriate mechanistic level. The predicted functional pathways and differences in the abundance of metabolite-producing taxa represent computational inferences of microbial functional potential rather than direct evidence of changes in metabolite concentrations. Shotgun-metagenomic functional profiling is an established approach for characterizing the metabolic potential of microbial communities [53], and large human studies have shown that microbial pathway abundances can be reproducibly associated with measured metabolites, even though pathway abundance is not equivalent to metabolic output [54]. Meta-analytic evidence likewise supports the robustness of microbiome–metabolome associations across studies [55], while pathway-centric approaches such as gutSMASH further demonstrate the value of genome-resolved prediction for identifying specialized microbial metabolic pathways [56]. Thus, the present metagenomic findings provide biologically informative evidence of altered microbial functional potential, while direct metabolomic measurement remains necessary to determine whether these signatures translate into changes in SCFAs or phenolic metabolite concentrations. Similarly, the present trial did not directly measure mucosal inflammation, intestinal barrier function, oxidative stress, visceral sensitivity, or neuroendocrine and metabolic biomarkers. The previously described anti-inflammatory, antioxidant, barrier-protective, and visceral analgesic mechanisms should therefore be regarded as literature-supported biological context rather than mechanisms directly demonstrated in our participants. Nevertheless, the consistency between the clinical improvements observed in the present trial and the effects previously demonstrated experimentally with WThE strengthens the biological plausibility of these mechanisms. The significant improvements in stress-, coping-, and disease-control-related FDDQL domains provide supportive clinical evidence of improved health-related well-being and a rationale for further investigation of potential gut–brain axis mechanisms using direct biomarker measurements.

Several limitations should be acknowledged. First, dietary intake was not systematically recorded during the study, as participants maintained their habitual diets without formal restrictions. Although diet may influence gastrointestinal symptoms and microbiota composition, the randomized, double-blind, placebo-controlled multicenter mitigates the potential confounding effect of individual dietary variability across treatment groups. Clinical evolution, symptoms, and compliance were assessed using validated questionnaires at standardized study visits. Although daily diaries could have provided more granular information, the standardized follow-up adequately captured the overall clinical and microbial changes observed during the intervention. Second, missing week-8 data were addressed using multiple imputation under a missing-at-random assumption. The robustness of the primary findings was further supported by complete-case (per-protocol) and tipping-point sensitivity analyses, which were consistent with the primary intention-to-treat results. Third, although the 8-week intervention was sufficient to detect clinically meaningful changes, longer studies are needed to determine the durability of these effects. The gut microbiome is a dynamic ecosystem influenced by diet, circadian rhythms, and other factors; therefore, the two sampling time points provide a standardized assessment of microbial changes over the intervention rather than a continuous characterization of microbial dynamics. Moreover, direct measurements of neuroendocrine, metabolic, inflammatory, or intestinal barrier biomarkers were not performed. The significant improvements in stress-, coping-, and disease-control-related FDDQL domains nevertheless provide supportive clinical evidence of improved health-related well-being and, together with the observed clinical and fecal microbial changes, provide a biologically plausible basis for further investigation of potential gut–brain axis mechanisms in future studies using direct biomarker measurements. Additional limitations include the predominantly female study population (80.7%), which may limit generalizability to men, and the exploratory nature of individual GSRS items and FDDQL domains, which were not adjusted for multiple comparisons. Accordingly, these outcomes should be interpreted as supportive and hypothesis-generating rather than as independent confirmatory findings. The fecal microbiome and predicted metagenomic profiles likewise provide evidence of specific microbial associations but do not directly measure intestinal barrier function, inflammatory signaling, neuroendocrine activity, or microbial metabolites. The anatomical limitation of fecal sampling should also be considered, as these data primarily reflect the distal colonic compartment and cannot establish where along the gastrointestinal tract the extract exerts its primary effects.

## 5. Conclusions

In conclusion, this study provides clinical evidence that 8 weeks of WThE supplementation reduced self-reported gastrointestinal symptom burden and improved selected health-related quality-of-life outcomes in adults with persistent gastrointestinal discomfort. The intervention was also associated with specific differences in fecal microbial taxa and predicted microbial pathways, without significant changes in global α- or β-diversity. The overall pattern is therefore more consistent with selective modulation of particular microbial taxa and functional potential than with broad microbiome remodeling. The chemically characterized phytochemical profile of WThE, particularly its rosmarinic acid content, alongside established evidence of human microbial metabolism of Lamiaceae phenolics, provides strong biological rationale for these observations. The convergence of clinical, microbiome, phytochemical, and preclinical evidence support a model in which WThE may influence gastrointestinal symptoms through complementary effects on microbial ecology, microbial metabolic potential, epithelial and immune pathways, smooth-muscle function, and visceral sensitivity. Future studies incorporating longer follow-up, multi-omics integration, and responder stratification will be important to confirm the durability of these effects and to further delineate the mechanisms underlying individual responses. In particular, targeted and untargeted metabolomics could establish whether the microbial functional signatures observed here are accompanied by changes in SCFAs and microbiota-derived phenolic metabolites, while simultaneous assessment of systemic inflammatory mediators, mucosal barrier integrity, visceral sensitivity, and neuroendocrine profiles could help clarify the proposed gut–brain and microbiota–host signaling pathways. Such integrated approaches would provide a more complete mechanistic understanding of the effects of WThE and formally evaluate the proposed prebiotic-like activity and bidirectional signaling pathways associated with gastrointestinal health.

## Figures and Tables

**Figure 1:**
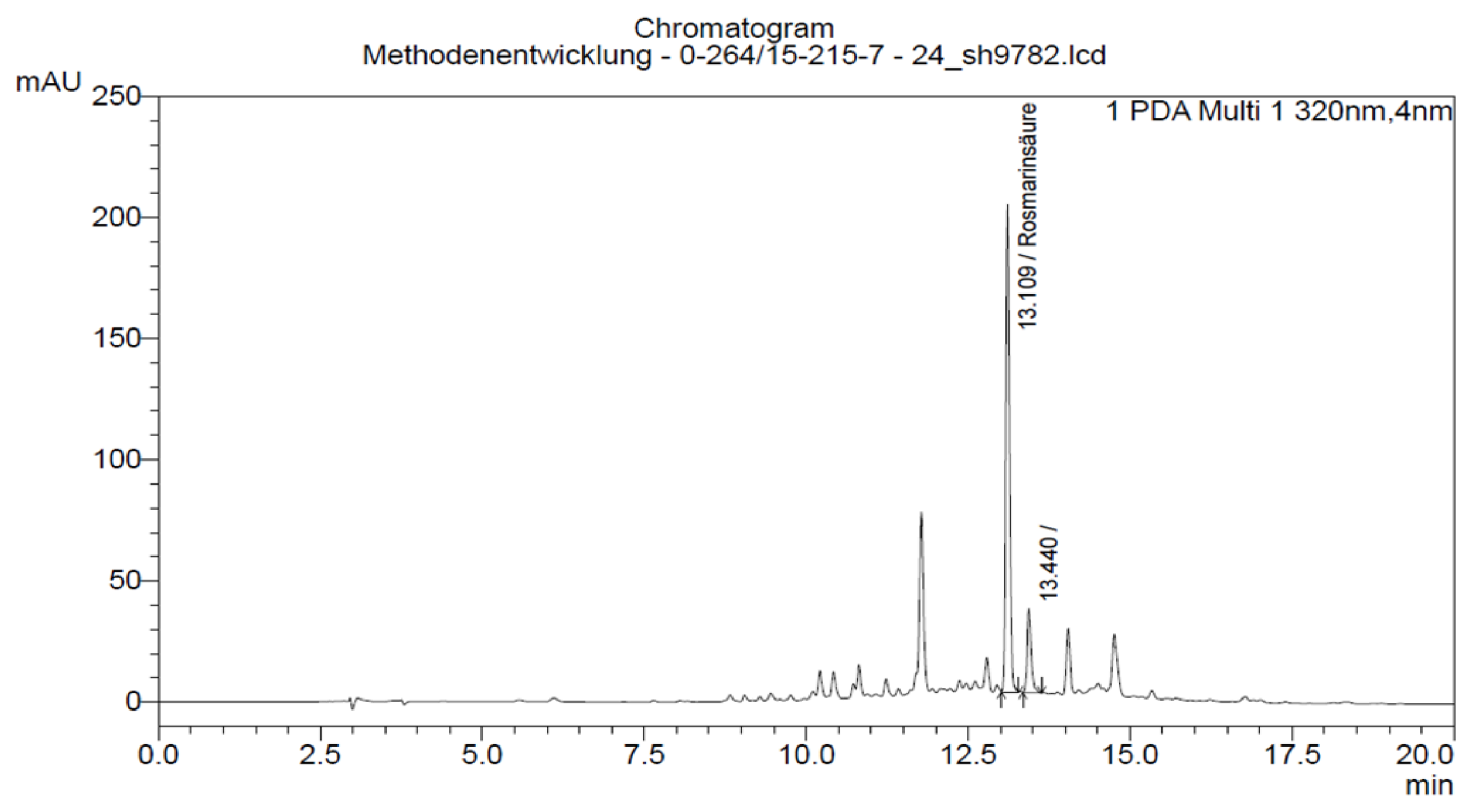
Quantitative determination of Rosmarinic acid (*Rosmarinsäure*) in dry extract of wild thyme dry extract (*Thymus serpyllum*) by high-performance liquid chromatography (HPLC). The HPLC analysis was performed with a UV-Vis detector at 320 nm. Separation was achieved on a RP18, 250 mm × 4.6 mm, 5 µm column with a mobile phase consisting of water:phosphoric acid (999:1 *v*/*v*) and acetonitrile: phosphoric acid (999:1 *v*/*v*) in a gradient mode. The HPLC chromatogram of batch 22000540 showed a rosmarinic acid content of 2.65% (*w*/*w*).

**Figure 2:**
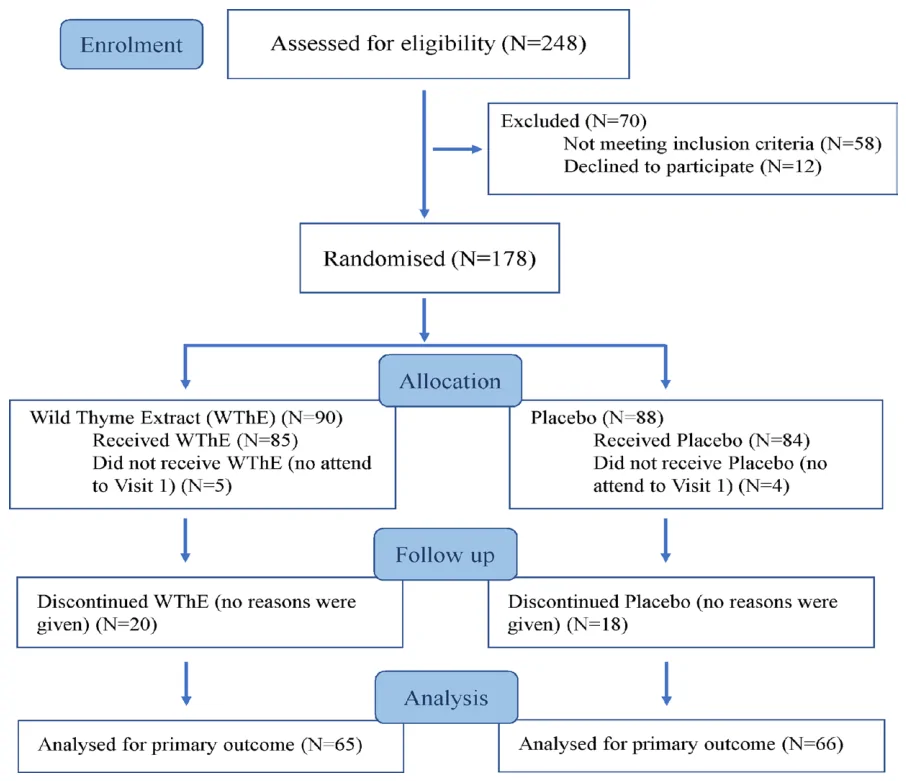
CONSORT of participant recruitment, eligibility, and study completion.

**Figure 3:**
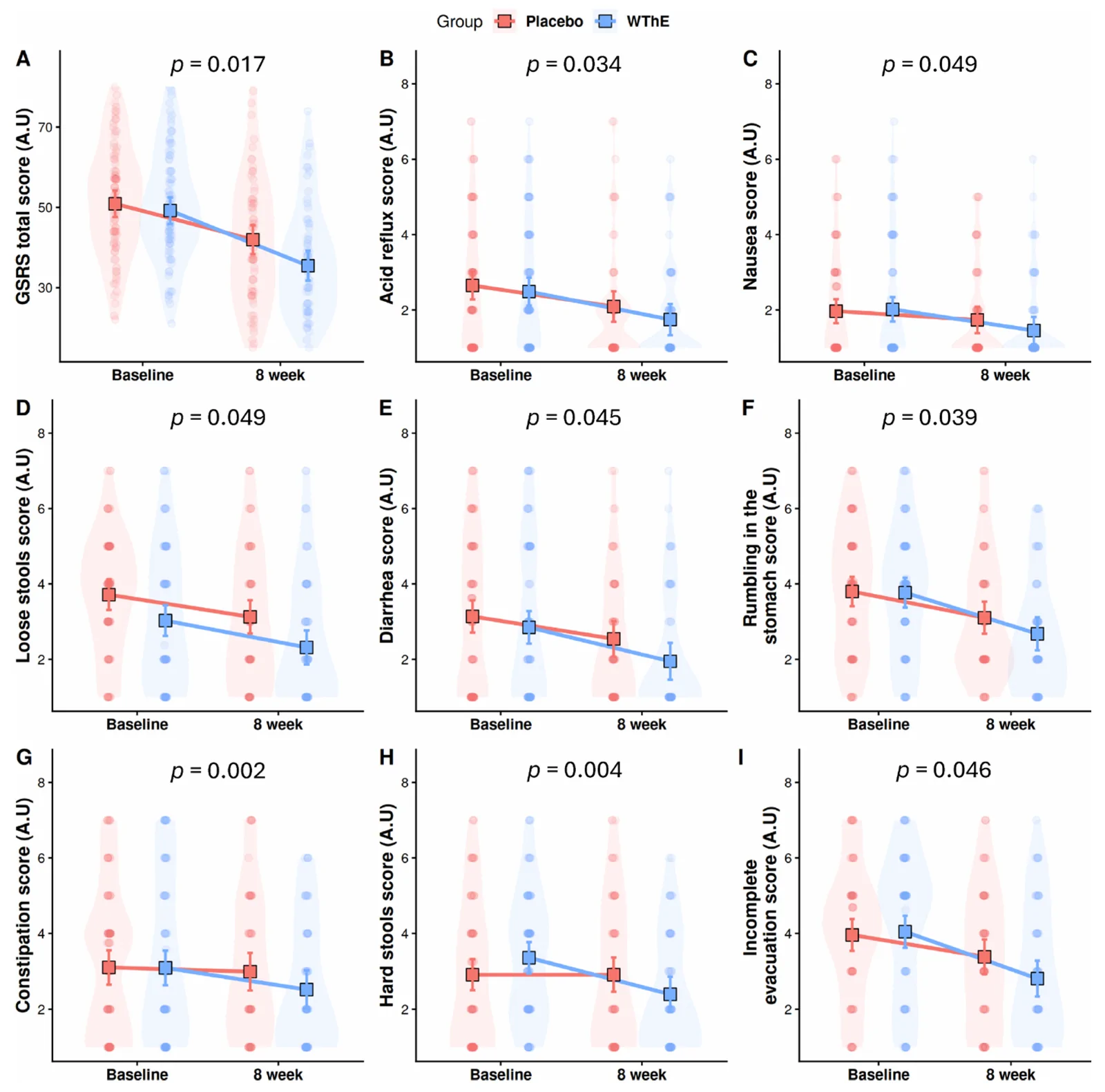
Changes in gastrointestinal symptoms assessed by the GSRS: (**A**) GSRS total score; (**B**) Acid reflux score; (**C**) Nausea score; (**D**) Loose stools score; (**E**) Diarrhea score; (**F**) Rumbling in the stomach score; (**G**) Constipation score; (**H**) Hard stools score; and (**I**) Incomplete evacuation score. Data are presented as mean ± SD. Statistical analysis: the *p*-values indicate between-group differences in changes from baseline to week 8. Abbreviations: GSRS, Gastrointestinal Symptom Rating Scale; SD, standard deviation; WThE, Wild Thyme extract.

**Figure 4:**
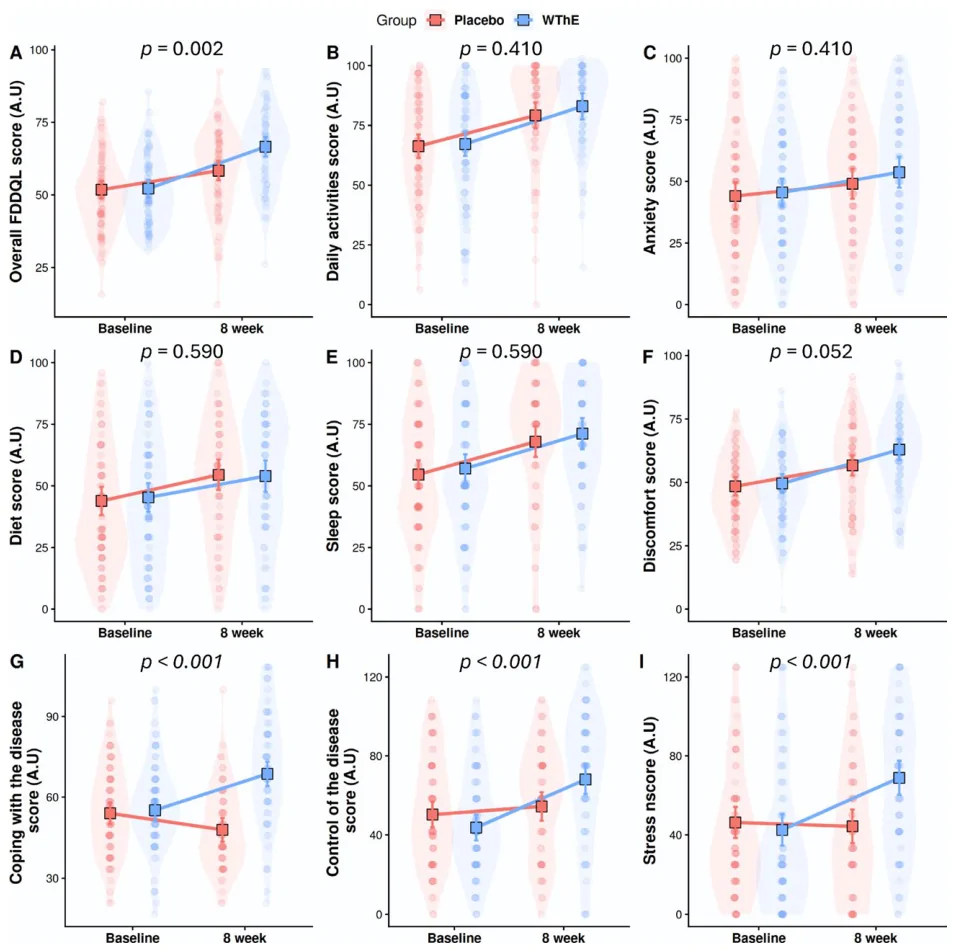
Changes in quality of life assessed by the Quality of Life Questionnaire for Functional Digestive Disorders (FDDQL): (**A**) Overall FDDQL score; (**B**) Daily activities score; (**C**) Anxiety score; (**D**) Diet score; (**E**) Sleep score; (**F**) Discomfort score; (**G**) Coping with disease score; (**H**) Control of disease score; and (**I**) Stress score. Data are presented as mean ± SD. The *p*-values indicate between-group differences in changes from baseline to week 8. Abbreviations: FDDQL, Functional Digestive Disorders Quality of Life Questionnaire; SD, standard deviation; WThE, Wild Thyme extract.

**Figure 5:**
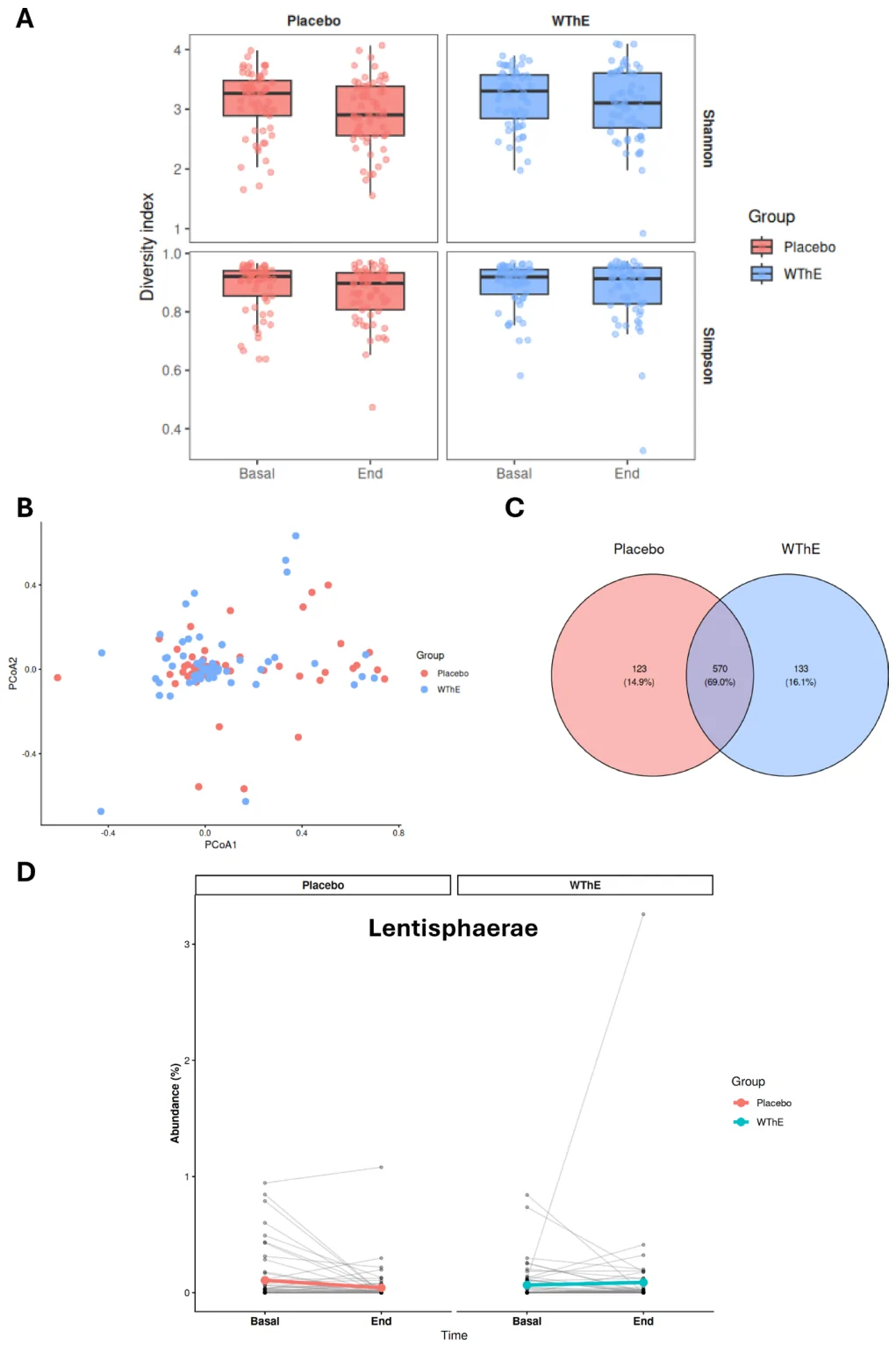
Fecal microbiota diversity, community structure, and *Lentisphaerae* abundance during the intervention. (**A**) Shannon and Simpson α-diversity indices of fecal microbiota at baseline and at the end of the 8-week intervention in the WThE and placebo groups. Data are shown as box plots with individual participant values. (**B**) Principal coordinates analysis (PCoA) based on Bray–Curtis dissimilarity, illustrating β-diversity and the overall structure of fecal microbial communities at baseline and at the end of the intervention in both study groups. (**C**) Venn diagram illustrating the microbial features shared between and specific to the WThE and placebo groups at the end of the intervention. (**D**) Relative abundance of the phylum *Lentisphaerae* at baseline and at the end of the intervention in the WThE and placebo groups. Thin grey lines represent individual participant trajectories, whereas thick coloured lines indicate group mean values. α-Diversity was analyzed using covariate-adjusted linear mixed-effects models, whereas differences in β-diversity were assessed by PERMANOVA. Differential abundance and prevalence of microbial taxa were analyzed using MaAsLin3. *p*-values were adjusted for multiple comparisons using the Benjamini–Hochberg false discovery rate (FDR) procedure; an FDR-adjusted *q*-value < 0.05 was considered statistically significant.

**Figure 6:**
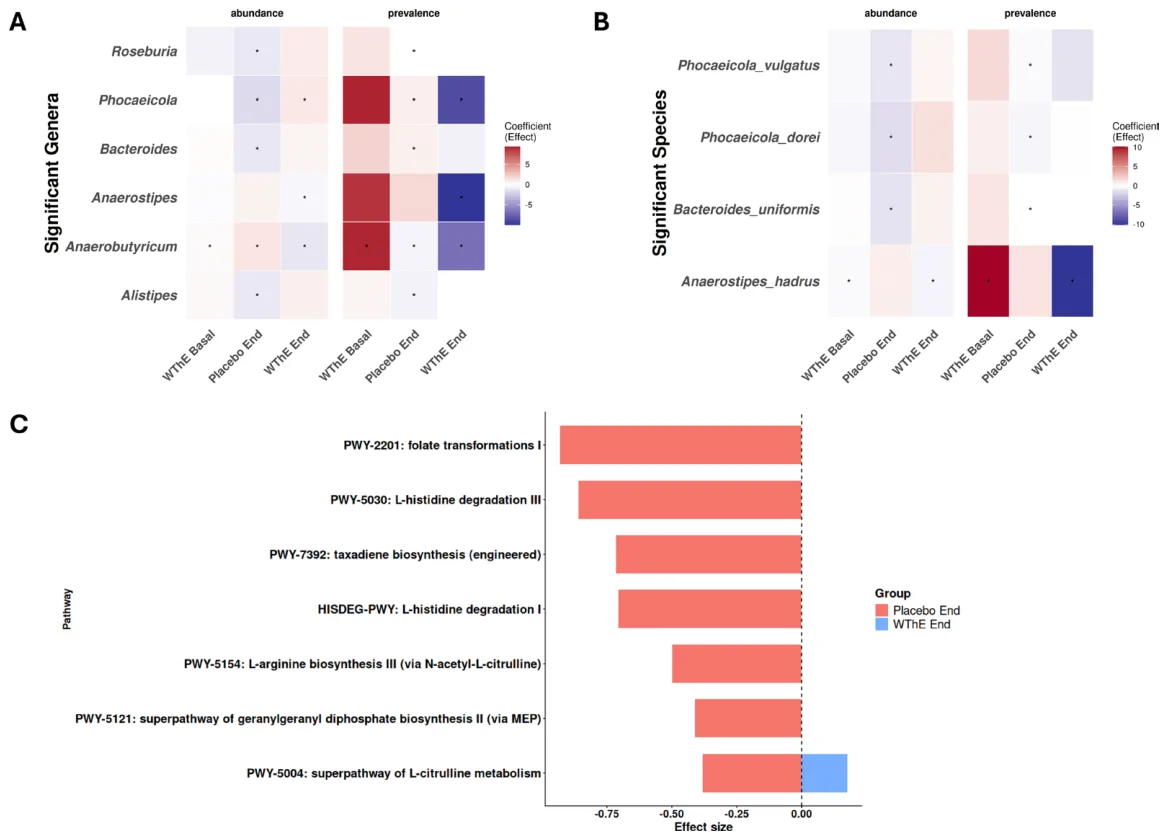
Differentially abundant bacterial taxa and microbial metabolic pathways following WThE supplementation. (**A**) Bacterial genera showing significant differences in abundance and/or prevalence across the study groups and time points. Heatmap cells represent model regression coefficients (effect sizes), with warm colours indicating positive and cool colours indicating negative coefficients. (**B**) Differential abundance and prevalence of the bacterial species that changed significantly after the intervention, displayed as in (**A**). (**C**) Effect sizes of HUMAnN-derived microbial metabolic pathways showing differential representation between study groups at the end of the intervention. Differential abundance, prevalence, and functional pathway analyses were performed using MaAsLin3, adjusting for age, sex, body mass index, and study site and incorporating subject-level random effects. *p*-values were corrected for multiple comparisons using the Benjamini–Hochberg FDR procedure. Asterisks indicate statistical significance (* *q* < 0.05).

**Figure 7:**
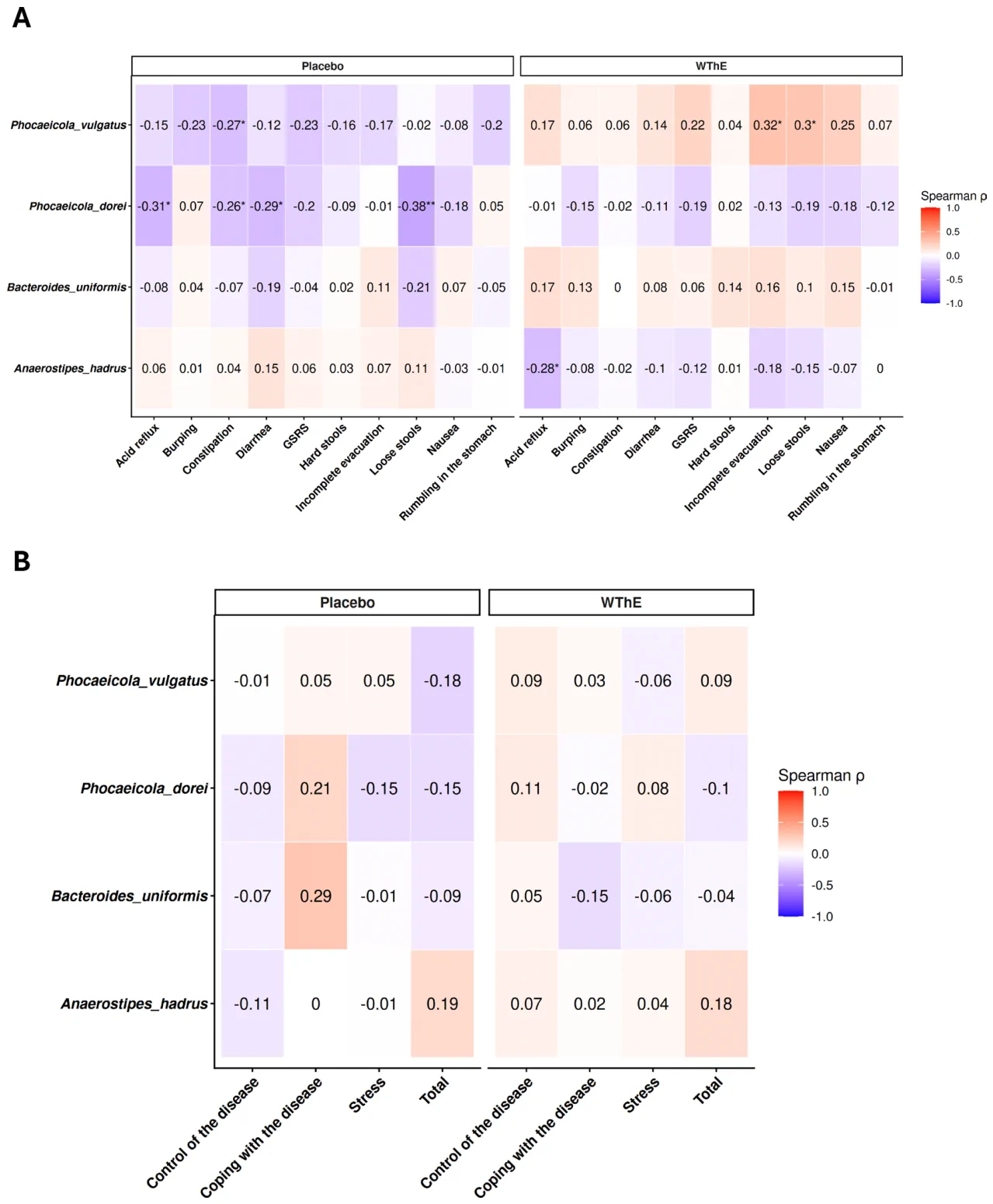
Correlation analysis between key bacterial species and clinical outcome measures (GSRS and QoL scores). (**A**) Spearman correlation analyses assessing the associations between the relative abundance of the most relevant bacterial species and gastrointestinal symptoms (GSRS) at the end of the intervention. (**B**) Spearman correlation analyses evaluating the associations between the relative abundance of the most relevant bacterial species and quality-of-life (QoL) scores at the end of the intervention. Analyses were stratified by treatment group (placebo vs. WThE) to identify group-specific patterns, and only bacterial species showing significant differential abundance between groups were included. Spearman’s rank correlation coefficients (ρ) and the corresponding Benjamini–Hochberg FDR-adjusted *q*-values are shown. * *q* < 0.05; ** *q* < 0.01.

**Table 1:** Baseline clinical and biochemical characteristics of the participants.

Characteristic	WThE *n* = 90 ^1^	Placebo *n* = 88 ^1^	Overall *n* = 178 ^1^
Age, years	44.00 (14.00)	42.10 (14.90)	43.10 (14.40)
Gender			
Female	70 (79.50%)	72 (81.80%)	142 (80.7%)
Male	18 (20.50%)	16 (18.20%)	34 (19.3%)
BMI, kg/m^2^	24.20 (3.70)	23.70 (3.60)	24.00 (3.60)
Fasting glucose, mg/dL	81.40 (12.30)	80.70 (16.70)	81.00 (14.70)
Serum creatinine, mg/dL	0.80 (0.10)	0.80 (0.20)	0.80 (0.10)
ALT, U/L	21.80 (9.90)	19.40 (11.80)	20.60 (10.90)
GGT, U/L	22.50 (12.20)	21.50 (10.80)	22.00 (11.50)
Serum Iron, µg/dL	98.30 (34.50)	93 (36.40)	95.90 (35.20)
Thyrotropin, µU/mL	2.10 (0.90)	2.20 (1.40)	2.10 (1.20)
Cholesterol, mg/dL	196.20 (37.20)	199.50 (40.50)	197.80 (38.70)
HDL, mg/dL	62.30 (14.60)	63.00 (15.00)	62.60 (14.70)
LDL, mg/dL	124.00 (41.80)	127.70 (32.80)	125.70 (37.80)
TGC, mg/dL	88.40 (56.50)	86.30 (53.50)	87.40 (54.80)

^1^ Mean (SD); *n* (%). WThE, *n* = 90; placebo, *n* = 88; overall, *n* = 178 randomized participants. Abbreviations: BMI, body mass index; ALT, alanine aminotransferase; GGT, gamma-glutamyltransferase; HDL, high density lipoprotein; LDL, low density lipoprotein; TGC, triglycerides.

**Table 2:** Intention-to-treat analysis of change from baseline to week 8 in gastrointestinal symptoms assessed by the GSRS according to treatment group.

Outcome ^a^	WThE (*n* = 90) ^b^	Placebo (*n* = 88) ^b^	Mean Difference (95% CI)	*p*-Value
**Primary outcome**				
GSRS score	−16.46 (−19.77 to −13.14)	−9.74 (−13.49 to −6.00)	−6.71 (−12.16 to −1.26)	0.017
**Secondary outcomes**				
*Reflux*				
Acid reflux score	−0.94 (−1.19 to −0.69)	−0.56 (−0.83 to −0.29)	−0.38 (−0.74 to −0.03)	0.034
Heartburn score	−0.93 (−1.19 to −0.66)	−0.80 (−1.09 to −0.51)	−0.13 (−0.55 to 0.29)	0.547
*Abdominal pain*				
Nausea score	−0.69 (−0.99 to −0.38)	−0.27 (−0.61 to 0.06)	−0.41 (−0.82 to −0.00)	0.049
Hunger score	−1.14 (−1.47 to −0.81)	−0.78 (−1.07 to −0.48)	−0.36 (−0.82 to 0.10)	0.118
Upper abdominal pain score	−1.26 (−1.62 to −0.89)	−1.12 (−1.41 to −0.83)	−0.14 (−0.60 to 0.32)	0.552
*Diarrhea*				
Loose stools score	−0.98 (−1.39 to −0.56)	−0.16 (−0.71 to 0.40)	−0.82 (−1.48 to −0.16)	0.017
Diarrhea score	−0.99 (−1.38 to −0.61)	−0.44 (−0.83 to −0.05)	−0.55 (−1.10 to −0.01)	0.045
Urgent need to defecate score	−0.74 (−1.07 to −0.42)	−0.87 (−1.25 to −0.49)	0.12 (−0.41 to 0.66)	0.637
*Indigestion*				
Rumbling in the stomach score	−1.34 (−1.70 to −0.98)	−0.76 (−1.11 to −0.40)	−0.58 (−1.13 to −0.03)	0.039
Bloated score	−1.51 (−1.90 to −1.12)	−1.30 (−1.80 to −0.80)	−0.21 (−0.82 to 0.40)	0.500
Burping score	−1.25 (−1.57 to −0.93)	−0.73 (−1.12 to −0.35)	−0.52 (−1.00 to −0.04)	0.034
Gas/Flatus score	−1.50 (−1.89 to −1.11)	−1.04 (−1.57 to −0.50)	−0.46 (−1.09 to 0.17)	0.146
*Constipation*				
Constipation score	−0.84 (−1.25 to −0.43)	0.04 (−0.37 to 0.46)	−0.89 (−1.44 to −0.33)	0.002
Hard stools score	−1.02 (−1.44 to −0.60)	−0.23 (−0.66 to 0.20)	−0.79 (−1.33 to −0.26)	0.004
Incomplete evacuation score	−1.30 (−1.73 to −0.86)	−0.72 (−1.14 to −0.30)	−0.57 (−1.13 to −0.01)	0.046

^a^ Change in score from baseline to week 8. ^b^ *n* denotes randomized participants included in the intention-to-treat analysis (WThE *n* = 90; placebo *n* = 88). Observed week-8 outcome data were available for 65 WThE and 66 placebo participants; model estimates were obtained using multiple imputation. Abbreviations: GSRS, gastrointestinal symptom rating scale.

**Table 3:** Intention-to-treat analysis of change from baseline to week 8 in health-related quality of life domains assessed by the FDDQL according to treatment group.

Domains ^a^	WThE (*n* = 90) ^b^	Placebo (*n* = 88) ^b^	Mean Difference (95% CI)	*p*-Value
Overall score	14.40 (11.31 to 17.5)	6.54 (3.47 to 9.60)	7.87 (3.56 to 12.17)	0.002
Daily activities	15.79 (10.84 to 20.74)	12.94 (8.04 to 17.85)	2.85 (−4.05 to 9.75)	0.410
Anxiety	8.18 (2.88 to 13.48)	4.97 (−0.28 to 10.22)	3.21 (−4.17 to 10.60)	0.410
Diet	8.68 (3.62 to 13.73)	10.61 (5.61 to 15.61)	−1.94 (−8.98 to 5.10)	0.590
Sleep	14.13 (8.75 to 19.52)	13.34 (8.01 to 18.67)	0.79 (−6.71 to 8.29)	0.590
Discomfort	13.36 (9.64 to 17.08)	8.23 (4.54 to 11.91)	5.13 (−0.05 to 10.31)	0.052
Coping with the disease	13.49 (9.26 to 17.71)	−6.12 (−10.30 to −1.93)	19.60 (13.72 to 25.49)	<0.001
Control of the disease	24.31 (16.89 to 31.72)	4.10 (−3.26 to 11.46)	20.21 (9.87 to 30.54)	<0.001
Stress	26.31 (19.06 to 33.56)	−1.96 (−9.14 to 5.21)	28.27 (18.18 to 38.36)	<0.001

^a^ Change in score from baseline to week 8. ^b^ *n* denotes randomized participants included in the intention-to-treat analysis (WThE *n* = 90; placebo *n* = 88). Observed week-8 outcome data were available for 65 WThE and 66 placebo participants; model estimates were obtained using multiple imputation.

## Data Availability

The datasets supporting the findings of this study are openly available in Figshare (DOI: https://doi.org/10.6084/m9.figshare.32727798). Due to ethical restrictions and patient privacy concerns, the datasets generated and analyzed during the current study cannot be made openly available. De-identified or aggregated data may be provided by the corresponding author upon reasonable request, subject to approval by the institutional ethics committee. Further information and requests for data resources and reagents can be directed to and will be fulfilled by Jorge Garcia-Garcia (jgarcia.51@ugr.es) and M. Elena Rodriguez-Cabezas (merodri@ugr.es).

## Supplementary Materials

The following supporting information can be downloaded at https://www.mdpi.com/article/10.3390/medsci14060611/s1, Figure S1: Thin-Layer Chromatography (TLC) of dry extract of Wild Thyme (*Thymus serpyllum*) according to the European Pharmacopoeia’s Monograph Wild Thyme; Figure S2: Qualitative comparison of Wild Thyme (*Thymus serpyllum*) and Thyme (*Thymus vulgaris/Thymus zygis*) by TLC on flavonoids acc. to European Pharmacopoeia’s Monograph Wild Thyme; Figure S3: Qualitative comparison of aqueous extracts of Wild Thyme (*Thymus serpyllum*) and Thyme (*Thymus vulgaris/Thymus zygis*) by TLC on polar constituents; Figure S4: Quantitative determination of Thymol and Carvacrol in Wild Thyme (*Thymus serpyllum*) by gas chromatography (GC) acc. to European Pharmacopeia Monography Thyme; Figure S5: Qualitative comparison of methanolic extracts of Wild thyme (*Thymus serpyllum*) and Thyme (*Thymus vulgaris/Thymus zygis*) by Liquid chromatograph with high-resolution mass spectrometry (LC-HRMS; Acquity UPLC system (Waters); hybrid LTQ Orbitrap Discovery mass spectrometer; heated ESI ion source; Figure S6: Tipping-point analysis for the GSRS total score; Figure S7: Relative abundance at phylum level; Figure S8: Relative abundance at genus level; Figure S9: Relative abundance at species level; Table S1: Per-protocol analysis of change from baseline to week 8 in gastrointestinal symptoms assessed by the GSRS according to treatment group; Table S2: Venn diagram and list of group-specific bacterial taxa; Tables S3–S6: MaAsLin3 results at phylum, genus, species, and HUMAnN functional pathway levels; Tables S7 and S8: Spearman correlation analyses between specific bacterial taxa and GSRS and QoL questionnaire scores. The Tables S2–S8 are openly available in Figshare (DOI: https://doi.org/10.6084/m9.figshare.32727798).

## Abbreviations

The following abbreviations are used in this manuscript:

ALT	Alanine aminotransferase
BMI	Body mass index
FDDQL	Functional Digestive Disorders Quality of Life Questionnaire
FC	Fold change
FDR	False discovery rate
GGT	Gamma-glutamyl transferase
GMP	Good Manufacturing Practice
GSRS	Gastrointestinal Symptom Rating Scale
HACCP	Hazard Analysis and Critical Control Points
HDL	High-density lipoprotein
ISAPP	International Scientific Association for Probiotics and Prebiotics
ISO	International Organization for Standardization
LDL	Low-density lipoprotein
NCT	National Clinical Trial
QC	Quality control
SD	Standard deviation
SCFA	Short-chain fatty acid
SMS	Shallow metagenomic sequencing
SPSS	Statistical Package for the Social Sciences
T0	Baseline (pre-intervention)
T8	Week 8 (end of study)
WThE	Wild Thyme extract
