# Supplementary material for: Thymus serpyllum Extract Improves Gastrointestinal Symptoms and Modulates Fecal Gut Microbiome Composition in a Multicenter, Randomized, Placebo-Controlled Clinical Trial
Source: Med Sci (Basel). 2026 Sep 28;14(6):611. doi: 10.3390/medsci14060611 (PMC13707104; doi:10.3390/medsci14060611)
Supplement: Supplementary file 1 [file medsci-14-00611-s001.zip › medsci-4520015-supplementary.pdf]

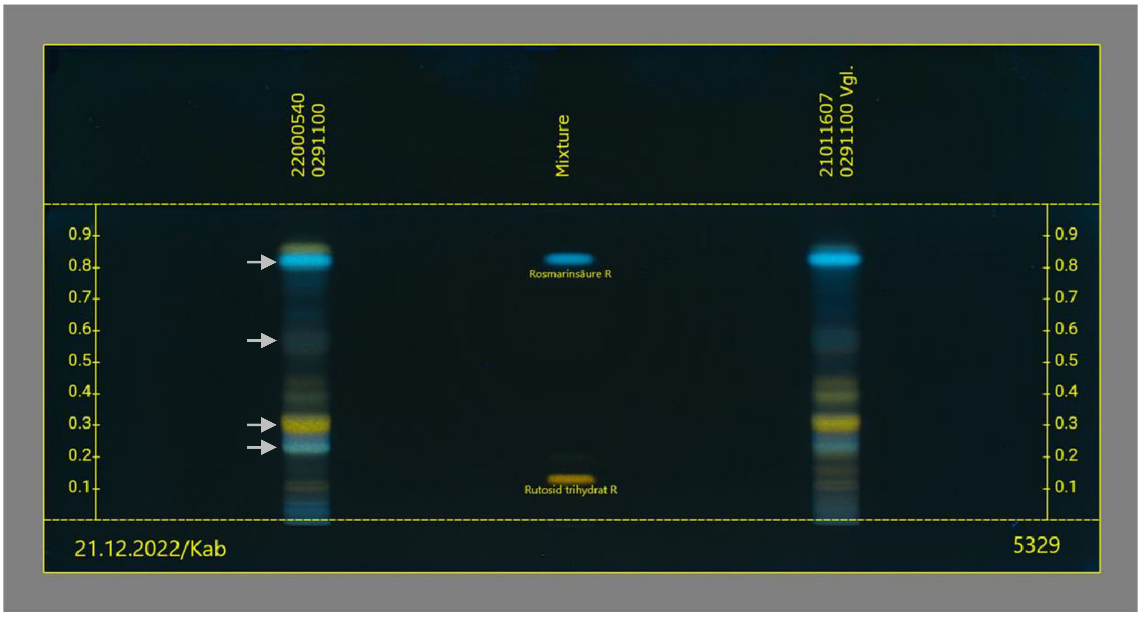

Supplementary Figure S1. Thin-Layer Chromatography (TLC) of dry extract of Wild Thyme (*Thymus serpyllum*) acc. to European Pharmacopoeia's Monograph Wild Thyme. The fingerprint on flavonoids and polyphenols in the 360GUT® batch n. 22000540 was reproducible in comparison with a previous batch (n. 21011607) and complied with defined by European Pharmacopoeia's Monograph and as indicated by arrows: a green/blue fluorescent and a yellow zones in the lower third; blue fluorescent zones in the middle third; and the blue zone correspondent to Rosmarinic acid (*Rosmaninsäure R*) in the upper third. Rutoside (*Rutosid trihydrat R*) is also used as control in the reference solution.

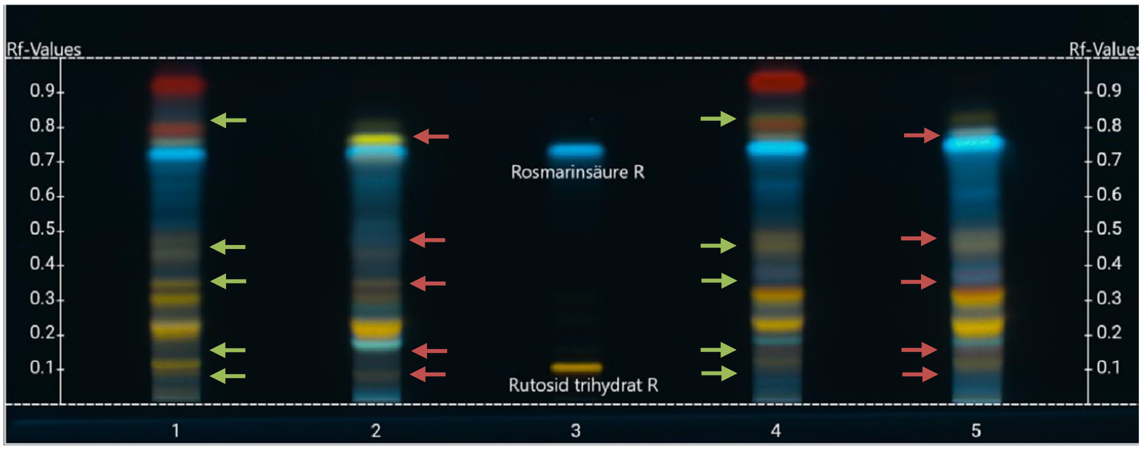

Supplementary Figure S2. Qualitative comparison of Wild thyme (*Thymus serpyllum*) and Thyme (*Thymus vulgaris*/*Thymus zygis*) by TLC on flavonoids acc. to European Pharmacopoeia's Monograph Wild Thyme. Sample 1 is Wild thyme methanolic extract; Sample 2 is Wild thyme aqueous extract; Sample 3 is Reference substances Rosmarinic acid (*Rosmarinsäure R*) and Rutoside (*Rutosid trihydratat R*); Sample 4 is Thyme methanolic extract, and Sample 5 is Thyme aqueous extract. The grey and orange arrows indicate the differences between the methanolic and aqueous extracts, respectively, what shows the characteristic TLC-fingerprints and distinguish the phytochemical profile of Wild Thyme from Thyme.

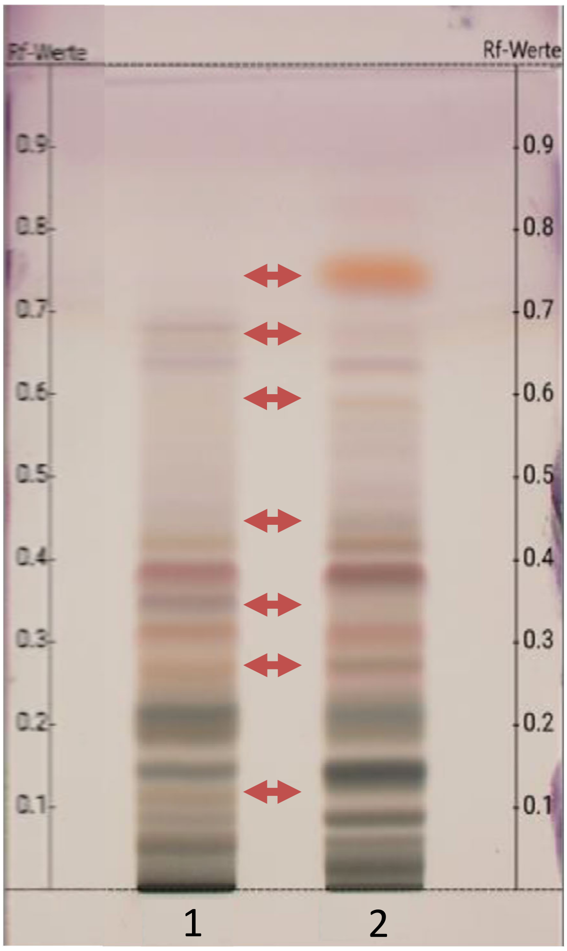

Supplementary Figure S3. Supplementary Figure S3. Qualitative comparison of aqueous extracts of Wild thyme (*Thymus serpyllum*, Sample 1) and Thyme (*Thymus vulgaris*/*Thymus zygis*, Sample 2) by TLC on polar constituents. Extract samples were extracted with methanol by sonication and the resulting solutions were applied to silica gel F254 plates. Chromatographic separation was performed using dichloroethane–acetic acid (98%)–methanol–water (50:25:15:10) as the mobile phase. After development and air drying, the plates were derivatized with anisaldehyde reagent R and examined under UV light. Orange arrows indicate differences between the phytochemical profiles that distinguish Wild Thyme from Thym**e.**

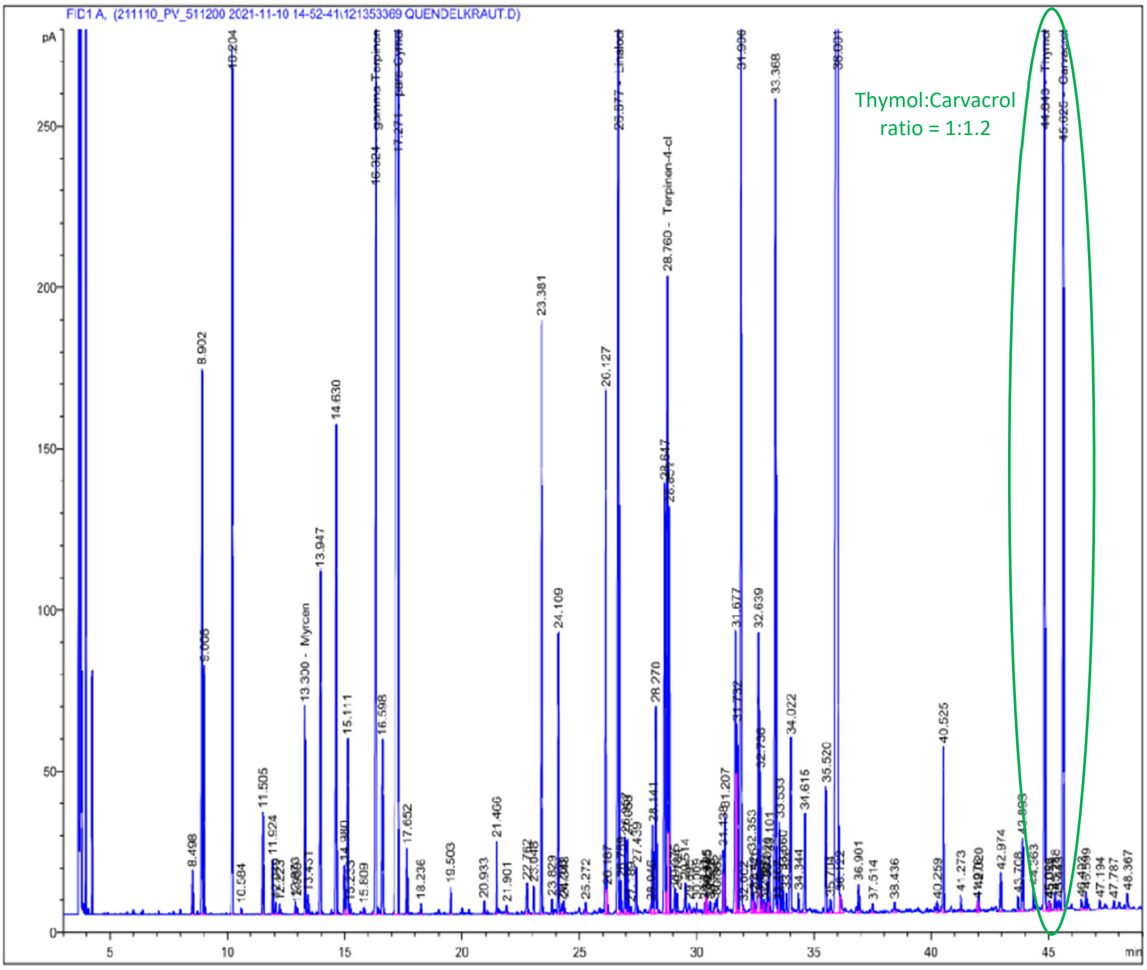

Supplementary Figure S4. Quantitative determination of Thymol and Carvacrol in Wild Thyme (*Thymus serpyllum*) by gas chromatography (GC) acc. to European Pharmacopeia Monography Thyme. Wild Thyme essential oil has shown the content (%Area) of Thymol and Carvacrol of 6.1% and 7.4%, respectively. This provides a ratio of 1:1.2 which is very distinguishable from Thyme (*Thymus vulgaris*/*Thymus zygis*) essential oil. The latter has shown the content (%Area) of Thymol and Carvacrol of 50.6% and 3.1%, respectively. This provides a ratio of 16:1.

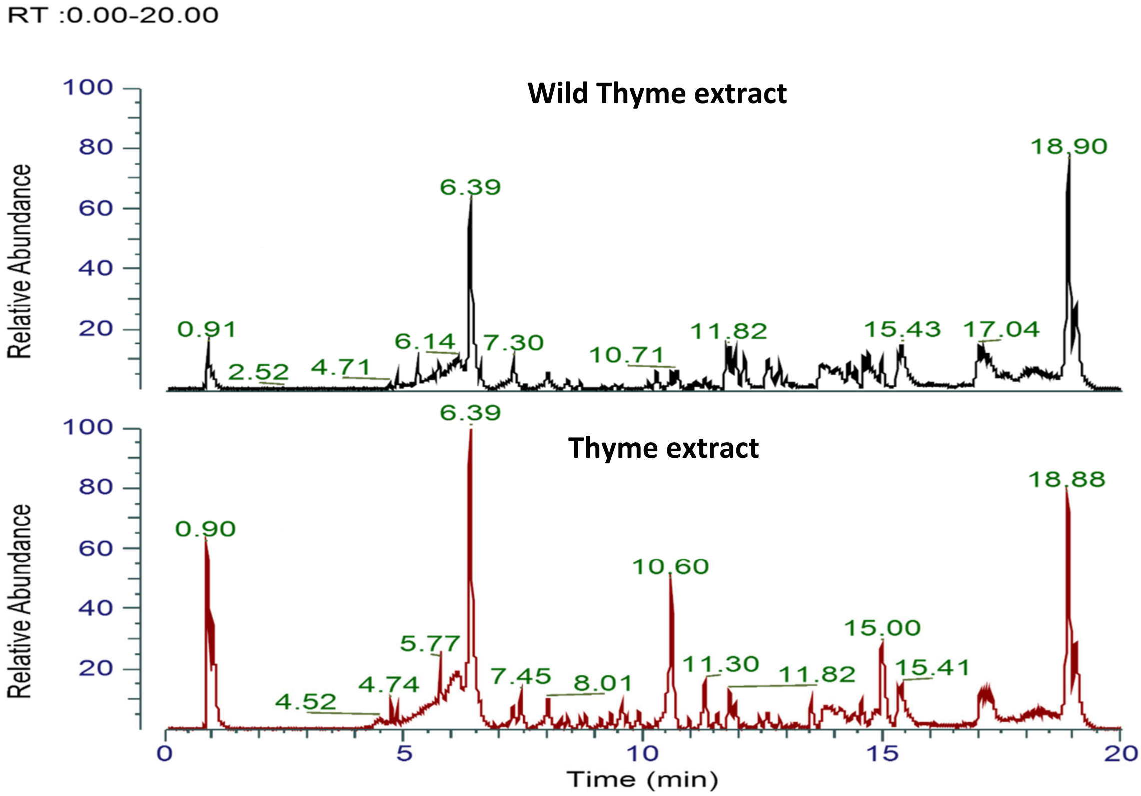

Supplementary Figure S5. Qualitative comparison of methanolic extracts of Wild thyme (*Thymus serpyllum*) and Thyme (*Thymus vulgaris*/*Thymus zygis*) by Liquid chromatograph with high-resolution mass spectrometry (LC-HRMS; Acquity UPLC system (Waters); hybrid LTQ Orbitrap Discovery mass spectrometer; heated ESI ion source. Gradient: H_2O with 0.1% formic acid (A) and acetonitrile (B) with 5% B (0–1min), 5–100% B (1–15min), 100% B (15–17min), 100–5% B (17–17.5min), 5% B (17.5–20min). Flow rate: 0.3 mL/min. Columns: C18; negative mode chromatograms). The results have tentative identified more than 40 common substances, mainly phenolic acid and flavonoids, such as Quimic acid (0.9 Min; *m*/*z* 191.0561; $C_7H_{12}O_6$); Malic acid (0.9 Min; *m*/*z* 133.0142; $C_4H_6O_5$); Syringic acid (2.3 Min; *m*/*z* 197.0455; $C_9H_{10}O_5$); Caffeic acid (4.7 Min; *m*/*z* 179.0350; $C_9H_8O_4$); Rosmarinic acid (6.39 Min; *m*/*z* 359.0772; $C_{18}H_{16}O_8$), several derivatives of Apigenin; Luteolin and Quercetin. The presence of different flavonoids was identified at 5.34 min (*m*/*z* 447.0933 $C_{21}H_{20}O_1$) and at 5.62 min (*m*/*z* 463.0882 $C_{21}H_{20}O_{12}$) in Thyme and Wild Thyme extracts, respectively. Derivatives of Salvianolic acid K (*m*/*z* 555.1149 $C_{27}H_{24}O_{13}$) have been identified in both species, however, different isomers are contained at 6.24 min and 6.41 min in Thyme and Wild Thyme extracts, respectively.

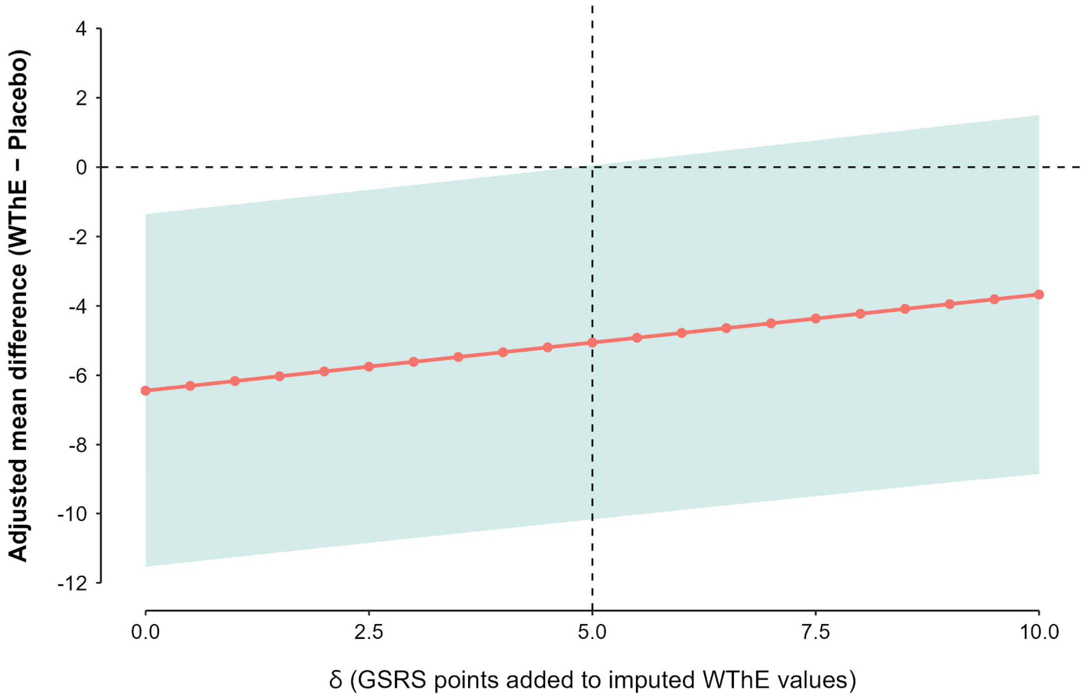

Supplementary Figure S6. Tipping-point analysis for the GSRS total score. The red line shows the adjusted between-group difference (WThE − placebo) under increasing penalties (δ) applied to imputed WThE values; shaded areas indicate 95% CIs. The effect remained statistically significant up to δ = 4.5 and lost significance at δ = 5.0. Across δ = 0–10, point estimates consistently favored WthE.

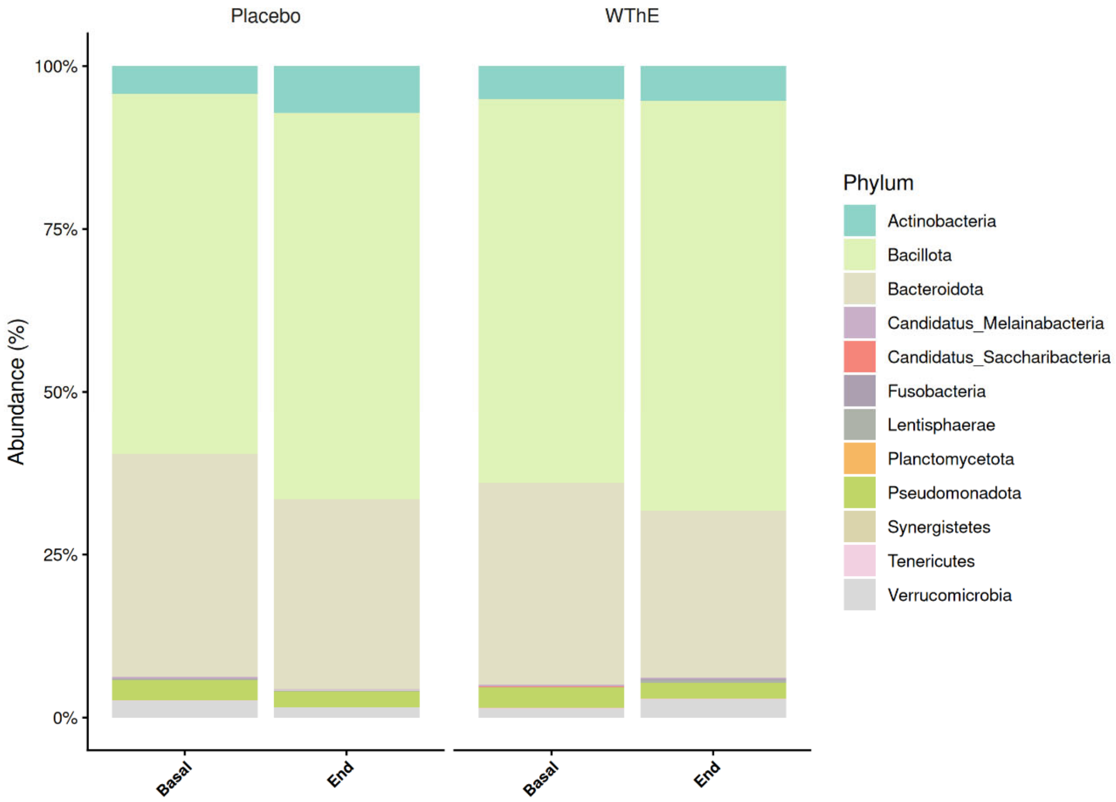

Supplementary Figure S7. Relative abundance at Phylum level. Representation of the detected phyla in both groups of study at basal and end point. Abundance is plotted as the mean abundance of each phylum.

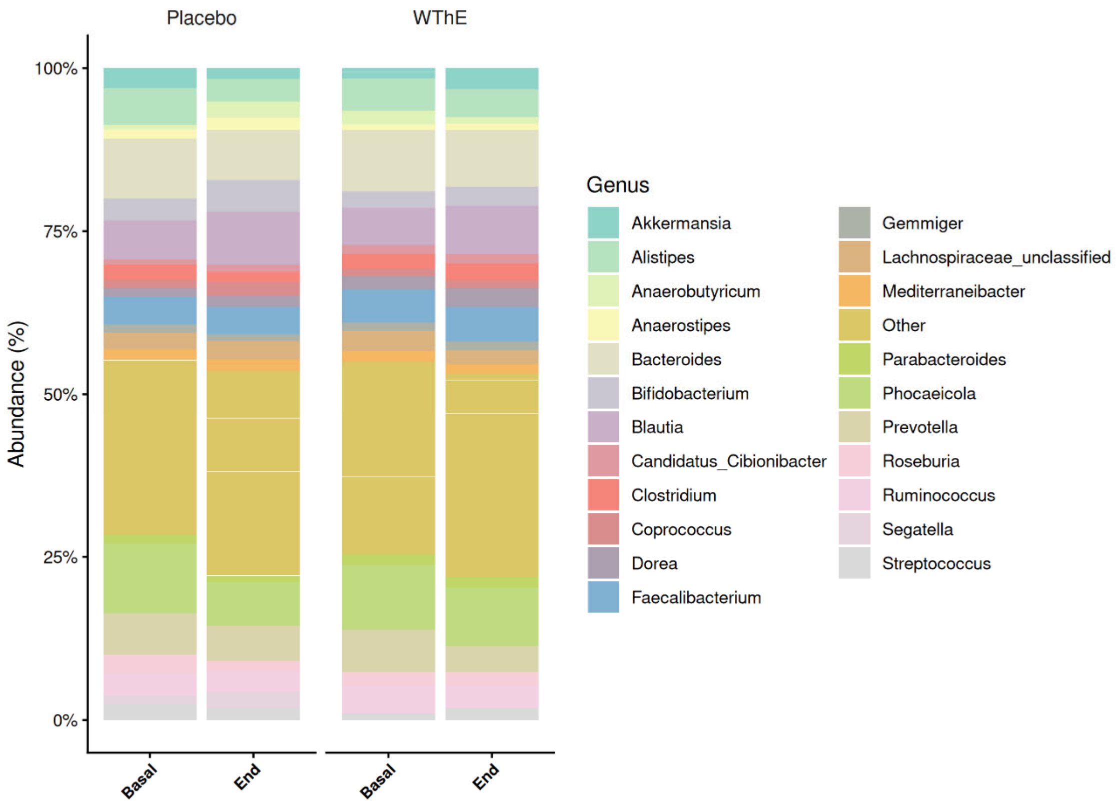

Supplementary Figure S8. Relative abundance at Genus level. Representation of the detected genus in both groups of study at basal and end point. Abundance is plotted as the mean abundance of each phylum. Taxa labeled as Other were those with an abundance and prevalence below 1% and 20%, respectively.

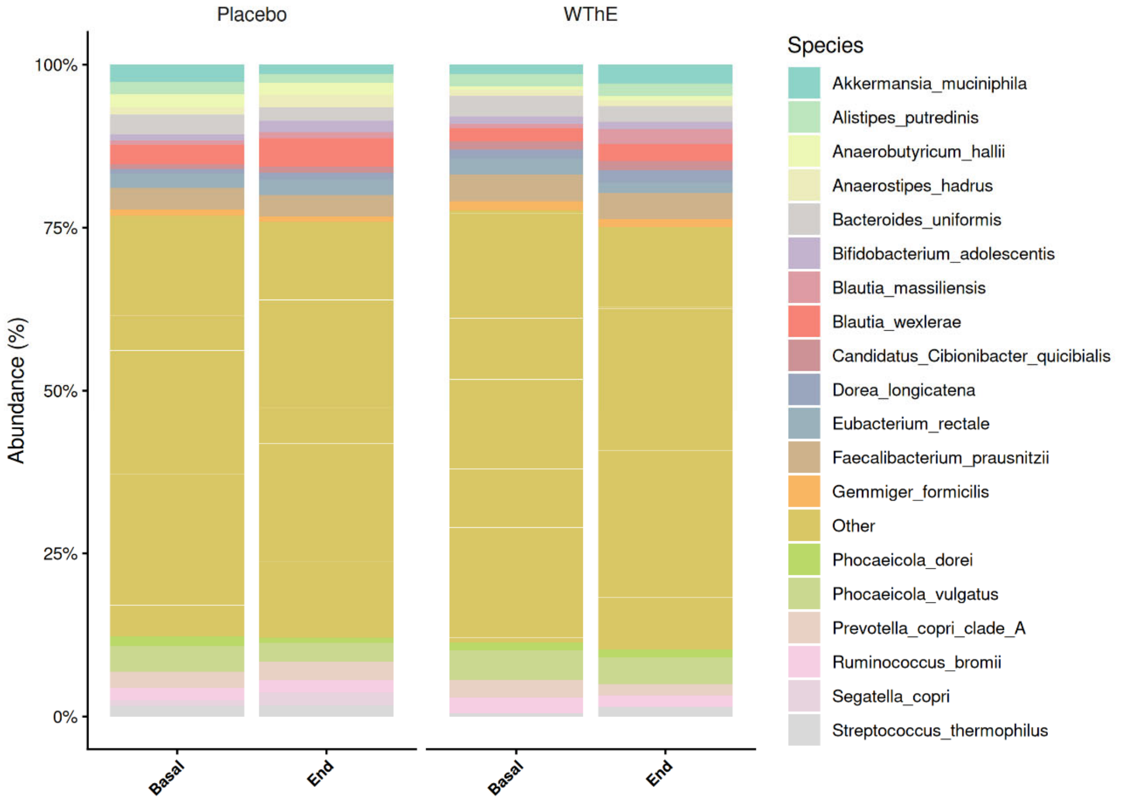

Supplementary Figure S9. Relative abundance at Species level. Representation of the detected species in both groups of study at basal and end point. Abundance is plotted as the mean abundance of each phylum. Taxa labeled as Other were those with an abundance and prevalence below 1% and 20%, respectively.

Supplementary Table S1: Per-protocol analysis of change from baseline to week 8 in gastrointestinal symptoms assessed by the GSRS according to treatment group.

Outcome[a]	WThE (N = 90)[b]	Placebo (N = 88)[b]	Mean Difference (95% CI)	P value
**Primary outcome**				
GSRS score	-15.87 (-19.13 to -12.61)	-9.13 (-12.36 to -5.89)	-6.75 (-11.34 to -2.15)	0.004
**Secondary outcomes**				
Reflux				
Acid reflux score	-0.96 (-1.24 to -0.69)	-0.53 (-0.81 to -0.26)	-0.43 (-0.82 to -0.04)	0.029
Heartburn score	-0.95 (-1.21 to -0.68)	-0.82 (-1.09 to -0.56)	-0.12 (-0.50 to 0.25)	0.517
Abdominal pain				
Nausea score	-0.68 (-0.96 to -0.39)	-0.24 (-0.53 to 0.04)	-0.43 (-0.84 to -0.03)	0.035
Hunger score	-1.12 (-1.42 to -0.83)	-0.71 (-1.01 to -0.42)	-0.41 (-0.82 to 0.01)	0.056
Upper abdominal pain score	-1.19 (-1.51 to -0.87)	-0.98 (-1.30 to -0.67)	-0.20 (-0.65 to 0.25)	0.376
Diarrhea				
Loose stools score	-0.98 (-1.39 to -0.57)	-0.31 (-0.71 to 0.10)	-0.67 (-1.25 to -0.10)	0.022
Diarrhea score	-0.97 (-1.36 to -0.59)	-0.42 (-0.80 to -0.04)	-0.56 (-1.10 to -0.01)	0.044
Urgent need to defecate score	-0.69 (-1.04 to -0.34)	-0.79 (-1.14 to -0.45)	0.10 (-0.39 to 0.60)	0.678
Indigestion				
Rumbling in the stomach score	-1.26 (-1.60 to -0.92)	-0.73 (-1.06 to -0.39)	-0.53 (-1.01 to -0.06)	0.029
Bloated score	-1.36 (-1.77 to -0.95)	-1.22 (-1.63 to -0.82)	-0.13 (-0.71 to 0.44)	0.648
Burping score	-1.20 (-1.55 to -0.86)	-0.72 (-1.07 to -0.38)	-0.48 (-0.97 to 0.01)	0.055
Gas/Flatus score	-1.54 (-1.94 to -1.15)	-0.99 (-1.38 to -0.61)	-0.55 (-1.10 to 0.00)	0.051
Constipation				
Constipation score	-0.75 (-1.18 to -0.33)	0.09 (-0.33 to 0.51)	-0.85 (-1.44 to -0.25)	0.005
Hard stools score	-0.95 (-1.35 to -0.56)	-0.20 (-0.59 to 0.20)	-0.76 (-1.32 to -0.19)	0.008
Incomplete evacuation score	-1.18 (-1.57 to -0.79)	-0.61 (-1.00 to -0.23)	-0.57 (-1.12 to -0.02)	0.042

[a] Change in score from baseline to week 8

[b] Estimated mean (95% CI)

Abbreviations: GSRS, gastrointestinal symptom rating scale.
